# Advances in Heterogeneous Catalysts for Lignin Hydrogenolysis

**DOI:** 10.1002/advs.202306693

**Published:** 2023-11-14

**Authors:** Zhensheng Shen, Chengxiang Shi, Fan Liu, Wei Wang, Minhua Ai, Zhenfeng Huang, Xiangwen Zhang, Lun Pan, Ji‐Jun Zou

**Affiliations:** ^1^ Key Laboratory for Green Chemical Technology of Ministry of Education School of Chemical Engineering and Technology Tianjin University Tianjin 300072 China; ^2^ Collaborative Innovative Center of Chemical Science and Engineering (Tianjin) Tianjin 300072 China; ^3^ Haihe Laboratory of Sustainable Chemical Transformations Tianjin 300192 China

**Keywords:** bio‐chemicals, heterogeneous catalysts, hydrogenolysis, lignin, synergistic effect

## Abstract

Lignin is the main component of lignocellulose and the largest source of aromatic substances on the earth. Biofuel and bio‐chemicals derived from lignin can reduce the use of petroleum products. Current advances in lignin catalysis conversion have facilitated many of progress, but understanding the principles of catalyst design is critical to moving the field forward. In this review, the factors affecting the catalysts (including the type of active metal, metal particle size, acidity, pore size, the nature of the oxide supports, and the synergistic effect of the metals) are systematically reviewed based on the three most commonly used supports (carbon, oxides, and zeolites) in lignin hydrogenolysis. The catalytic performance (selectivity and yield of products) is evaluated, and the emerging catalytic mechanisms are introduced to better understand the catalyst design guidelines. Finally, based on the progress of existing studies, future directions for catalyst design in the field of lignin depolymerization are proposed.

## Graphical abstract

In this review, factors affecting catalyst performance are systematically summarized based on the three most commonly used supports (carbon, oxides, and zeolites) in lignin hydrogenolysis. The catalytic performance is evaluated, and the emerging catalytic mechanisms are introduced to better understand the catalyst design guidelines. Finally, future directions for catalyst design and catalytic conversion of lignin are prospected.

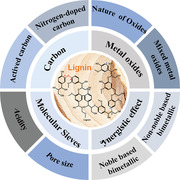

## 1 Introduction

With the progress of human beings and the development of social civilization, human demand for chemicals and fuels is increasing dramatically. The excessive consumption of fossil resources has led to the release of large amounts of greenhouse gases, causing problems such as global warming and environmental pollution.^[^ ^1^ ^]^ Therefore, how to use renewable resources innovatively and fully is of great significance to the future of sustainable development. Biomass, being a renewable resource, has garnered significant interest for its potential to be utilized in the production of chemicals, materials, and fuels with added value.^[^ ^2^ ^]^ Lignocellulose is a highly abundant form of biomass on our planet, consisting mainly of cellulose, lignin and hemicellulose. which is the most abundant organic hydrocarbon on earth resources, with the advantages of being renewable and “carbon neutral”.^[^ ^3^ ^]^

**Figure 1:**
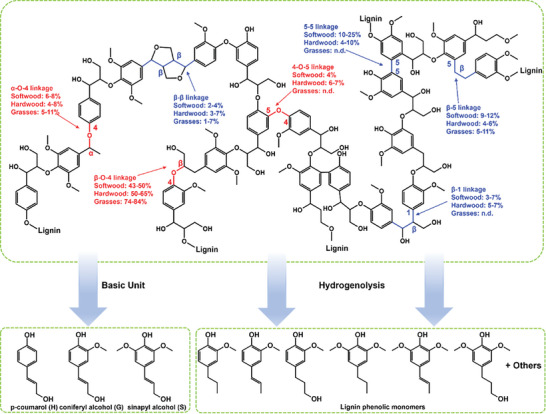
Lignin structure, basic units and phenolic monomers.

Cellulose and hemicellulose are complex polymers made up of sugar units connected by glycosidic bonds. Lignin is a biopolymer consisting of three main structural units: sinapyl alcohol (S), coniferyl alcohol (G), and p‐coumarol (H), linked together by C─C and C─O bonds (**Figure** **1**). The recalcitrant macromolecule structure plays an essential role in preventing microorganisms and moisture from damaging the protein matrix of cells, but it also seriously hinders its chemical utilization.^[^ ^4^ ^]^ Currently, the majority of biorefinery processes focus on using cellulose and hemicellulose fractions while disregarding the lignin fraction, which is a by‐product of pulping, by burning it as a low‐value fuel.^[^ ^1^, ^5^ ^]^ Nevertheless, lignin holds significant promise as an eco‐friendly resource for the creation of valuable products.

In recent years, great efforts have been made to develop efficient solutions for the conversion and utilization of lignin. Different techniques have been studied to improve the depolymerization of lignin, including biochemical,^[^ ^6^ ^]^ photocatalysis,^[^ ^3c^ ^]^ electrochemistry,^[^ ^7^ ^]^ reductive depolymerization,^[^ ^8^ ^]^ oxidative depolymerization,^[^ ^9^ ^]^ acid‐catalyzed depolymerization.^[^ ^10^ ^]^ Among many lignin depolymerization methods, reductive depolymerization enables the breakage of C─O─C bonds in lignin with relatively gentle conditions and inhibit the further condensation of lignin monomers, thereby obtaining high‐yield phenolic monomers and oligomers. It is important to note that a “lignin‐first” approach reductive catalytic fractionation (RCF) makes it possible to efficiently produce phenolic monomers, dimers, and oligomers directly from lignocellulose while preserving solid carbohydrate pulp. This pulp can be further transformed into fuels and chemicals, or utilized as feedstock for material applications. This process begins by extracting lignin from the lignocellulosic matrix, followed by catalytic degradation of lignin polymers and stabilization of phenolic compounds.^[^ ^11^ ^]^ RCF can be regarded as a more advanced reductive depolymerization method, so the case of RCF is also included in the discussion of lignin reductive depolymerization catalysts in this review.

In a typical lignin reductive depolymerization system, lignin or lignocellulose are treated at temperature of 180–300 °C and pressure of 0–5 MPa H_2_ (room temperature). The solvent is usually a polar alcohol solvent or other hydrogen‐donating solvent. Due to solvolysis, the lignin in the matrix is cleaved and depolymerized into small fragments. Some of these fragments interact with the catalyst and undergo hydrogenation to form stable phenolic monomers.^[^ ^2^, ^12^ ^]^ These monomers are widely used in various fields such as materials, energy, and medicine, etc.^[^ ^2^, ^13^ ^]^ Thus, enhancing the conversion of lignin and controlling the product selectivity are crucial factors in the hydrogenolysis reaction of lignin.

Heterogeneous catalysts have been widely applied in reductive depolymerization of lignin owing to their adjustable chemical/physical properties. The characterization of metals and supports and their interactions makes it easier to regulate catalytic activity towards high yield and high selectivity of the product as needed. Although there have been several reviews about lignin depolymerization in recent years,^[^ ^11^, ^14^ ^]^ they focused mainly on the method of lignin depolymerization or the process of lignin depolymerization without focusing on the structure of the catalyst, the role of metals and supports. To design catalysts with higher activity, it is necessary to review the structure and properties of the catalyst.

At present, the catalysts for lignin reductive depolymerization are mainly based on metal (such as Pd, Ru, Ni, etc. with excellent hydrogen dissociation properties) supported on different supports. The role of reactive metals is well understood, i.e., they mainly play the role of stabilizing the reactive intermediates, preventing the re‐polymerization of reactive monomers and increasing the monomer yield. However, the key role of catalyst supports in lignin depolymerization is still unclear. Different types of supports have different acidic and pore size structures and other physicochemical properties, which can significantly affect the yield and distribution of the products.

In this review, we divide the three most commonly used catalyst supports (carbon, oxide and zeolite) in lignin reduction depolymerization and systematically review the factors affecting catalyst performance (including the type of active metal, metal particle size, acidity, pore size, the nature of the oxide supports, and the synergistic effect of the metals). First, the classification of lignin as well as its structural characteristics are introduced (Section 2), which are the basis of lignin depolymerization as well as biomass refining. Then the application of carbon supported catalysts (Section 3), metal oxides supported catalysts (Section 4), molecular sieves supported catalysts (Section 5) in reductive depolymerization are reviewed separately. The catalytic performance (selectivity and yield of products) is evaluated, and the catalytic mechanisms that emerge are summarized. The synergistic effects of metal catalysts (Section 6) are also summarized, which provide ideas for further understanding of the design of lignin hydrogenolysis catalysts. Finally, the potential challenges and development opportunities of using heterogeneous catalysts for lignin depolymerization are prospected.

## 2 Classification of Lignin

Different degrees of modified lignin would be produced due to the different pulping methods in the industry. The degree of lignin modification seriously influences the depolymerization efficiency and product distribution. It is necessary to present the classification and structural characteristics of lignin to compare the performance of catalysts more clearly.

### 2.1 Natural Lignin

Natural lignin is widely present in various lignocellulosic biomasses, including softwoods, hardwoods, and herbs. It consists of three fundamental building blocks that are connected in a randomized manner by C─C bonds or C─O─C bonds (Figure 1).^[^ ^15^ ^]^ C─C bonds mainly include β‐β, 5‐5, β−5, and β−1; C─O─C bonds mainly include β─O─4, α─O─4, 4─O─5. The strength of the dissociation energy of these bonds is ranked as follows: β−5>5‐5>4‐O‐5>β─O─4>β−1>α─O─4. Due to different plant species and structures, the lignin content, monomer content, and linking bond content in different lignocellulosic biomasses are different. From the perspective of lignin structure, hardwood contains G and S‐type skeleton units, while softwood is composed of G‐type structural as primary bond between lignin structural units, the content of β─O─4 bond in hardwood (50%−65%) is higher than that in softwood (43%−50%). Lignin degradation efficiency and outcome are determined by the structure and connections of lignin units. S‐type monomers are more efficiently released from lignin macromolecules through degradation reactions.^[^ ^2^, ^8^, ^16^ ^]^ G‐type monomers are prone to polycondensation reactions.^[^ ^8^, ^17^ ^]^ The amount of degradable β─O─4 linkages is responsible for the maximum potential yield of monomers during the degradation of lignin, typically equal to the square of the β─O─4 content percentage.^[^ ^2^, ^18^ ^]^

### 2.2 Modified Lignin

Typically, technical pulping techniques like kraft, sulfite, alkaline, and klason processes aim to extract top‐notch cellulose from lignocellulose. These methods involve using harsh conditions, such as employing a significant amount of inorganic salts, alkalis, or acids to obtain lignin that has undergone significant structural modifications.^[^ ^19^ ^]^

Kraft lignin is acquired through a process of sulfide pulping and typically has a sulfur content of 1–3%, which is present in both inorganic and organic forms. As a result of the extensive pulping, the structure of kraft lignin is more intricate than other types of lignin, containing a higher number of C─C bonds.^[^ ^11a^ ^]^ This makes kraft lignin a particularly difficult substrate for catalytic processing, particularly for noble metal catalysts that are easily hindered by sulfur. Klason lignin is geted by dissolving hemicellulose and cellulose with 72% sulfuric acid, which also seriously affects the natural structure of lignin. The soda lignin exhibits slight structural changes during the pulping process, making it easier to undergo catalytic conversion compared with other technical lignins.^[^ ^20^ ^]^

Organosolv lignin is usually obtained by treating lignocellulose with organic solvents, such as methanol, ethanol, acetic acid, or mixtures with water, usually at temperatures of 140–220 °C. Compared with other methods, the organic solvent method reaction medium can effectively solubilize lignin without structural modification of lignin. The extraction process of organosolv pulping is more effective in maintaining the inherent structure of lignin. Unlike the sulfate and sulfite methods used in traditional pulping processes, the organic solvent fractionation method yields lignin with a low molecular weight (500–5000 Da) and no sulfur content.^[^ ^10^, ^21^ ^]^

The Björkman process consists of a thorough grinding step, followed by the extraction of the lignin using an organic/aqueous solvent. This process results in the production of milled‐wood lignin (MWL). MWL has a structure that closely resembles natural lignin, mainly because of the extraction being carried out under neutral pH and gentle conditions.

In studies of lignin hydrogenolysis, except for RCF, which uses lignocellulosic biomass as the raw material, most of the studies use organosolv lignin as the raw material because of its lower molecular weight and structural similarity to natural lignin. However, many studies also use industrial lignin such as alkali lignin and sulfate lignin as raw materials. Although its structure is challenging to depolymerize, it is still significant for the rational utilization of resources.

## 3 Carbon Supported Catalysts

### 3.1 Activated Carbon Supported Catalysts

Activated carbons are usually produced from woody biomass, agricultural waste, and coal through high‐temperature pyrolysis and activation, such as sawdust,^[^ ^22^ ^]^ coconut shells, fruit pits and peels,^[^ ^23^ ^]^ peat^[^ ^24^ ^]^ and bituminous coal.^[^ ^25^ ^]^ Supports made of activated carbons offer several advantages, including affordability, resistance to acids and alkalis, well‐developed pore structure, a significant surface area, and exceptional adsorption capabilities.^[^ ^26^ ^]^ In addition, the combustion of carbon support combustion can quickly recover the noble metals supported on activated carbon. The properties of the catalyst will be influenced by the pore structure, specific surface area, and surface functional groups of activated carbon. Physical and chemical treatment methods can modify these parameters of the carbon support. The catalyst has a broader range of adjustment and adaptation, so the application of activated carbons as the support is increasingly widespread. Due to the excellent properties of activated carbon supports, it has been widely used in lignin catalytic depolymerization, including noble metal catalysts, transition metal catalysts, etc. Activated carbon supports usually only play a role in dispersing active metals during the lignin hydrogenolysis, and the catalytic performance are controlled by the active metals. **Table** **1** summarizes the recent studies in the hydrogenolysis of lignin or lignocellulose over carbon‐supported catalysts.

**Table 1:** Summary of lignin depolymerization catalyzed by carbon‐based catalysts.

	Substrate	Catalyst	Solvent	Temperature [°C]	Time [h]	Pressure /[MPa H_2_]	Yield [wt%]	Main product	Ref
1	Birch	Pd/AC	Methanol	200	9	3	40	4‐n‐propylsyringol and 4‐n‐propylguaiacol	[54]
2	Poplar wood	Pd/AC	Methanol/water (7:3)	200	3	2	44	Phenolic monomer	[55]
3	Organosolv lignin	Pd/AC	Methanol	250	5	3	2.6	Phenolic monomer	[56]
4	Acetonesolv lignin	Pd/AC	Dioxane	200	24	0.1	15	Phenolic monomer	[57]
5	Alkali lignin	Pd/AC+CrCl_3_	Methanol	260	5	4	28.5	Phenolic monomer	[58]
6	Organosolv lignin	Pd/AC +CrCl_3_	Methanol	280	5	4	20	Guaiacols, Phenols	[59]
7	Poplar wood	Pd/AC+ZnCl_3_	Methanol	225	12	3.4	40	Phenolic monomer	[60]
8	Poplar wood	Pd/AC+H_3_PO_4_	Methanol	200	3	2	42	Phenolic monomer	[61]
9	Bagasse	Pd/AC+H_4_SiW_12_O_40_	Isopropanol	170	5	–	34.91	4‐ethylphenol, 4‐ethylguaiacol	[62]
10	Birch wood sawdust	Pd/AC+Yb_III_‐triflate	Methanol	180	2	3	43	Monomer	[63]
11	Aerobic oxidation pretreatment of lignin	Ru/AC	Formic acid	110	24	–	8.24	Phenolic monomers	[64]
12	Eucalyptus sawdust	Ru/AC	Butanol/water (1:1)	200	2	3	49	4‐n‐propylsyringol and 4‐n‐propylguaiacol	[33]
13	Cornstalk hydrolysis residue	Ru/AC	Ethyl acetate/H_2_O	260	5	N_2_	42.7	Aromatics	[65]
14	Concentrated acid hydrolysis lignin	Ru/AC	Methanol/formic acid	350	60	1	6	Phenolic monomer	[34c]
15	Soda lignin	Ru/AC	Ethanol	350	0.67	3	3.4	Methylated‐, and ethylated phenols	[34b]
17	Organosolv lignin	Ru/AC+NaOH	Isopropanol: water = 7:3	220	1	2	21	Monomeric	[34a]
18	Lignosulfonate	Pt/C+CrCl_3_	Methanol	280	5	3	9.2	Monomers	[66]
19	Birch sawdust	Pt/C+CrCl_3_	Methanol/water (7:3)	190	6	–	36.9	4‐n‐propylsyringol and 4‐n‐propylguaiacol	[67]
20	Alkali lignin	Rh/C	Ethanol/water	250	3	3	2.9	Phenolic monomer	[68]
21	Birch sawdust	Ni/C	Methanol	200	6	0.1	45	Phenolic monomer	[40c]
22	Poplar lignin	Ni/C	Methanol	225	3	3	36.8	Phenolic monomer	[69]
23	Miscanthus	Ni/C	Methanol	225	12	3.5	68	4‐n‐propylsyringol and 4‐n‐propylguaiacol	[70]
24	Candlenut	Ni/C	Methanol	250	3	3	8	4‐propylbenzene‐1,2‐diol, 4‐Propenylbenzene‐1,2‐diol	[71]
25	Oak wood	Ni@Al_2_O_3_/AC	Methanol	190	3	3	23.4	4‐n‐propyl guaiacol, 4‐npropenyl guaiacol,	[72]
26	Organosolv lignin	Ni/LCNF	Ethanol/water (1:1)	300	5	10	7	Phenols	[73]
27	Organosolv poplar lignin	NiCu/C	Ethanol/isopropanol	270	4	N_2_	63.4	Propylguaiacol, isoeugenol	[74]
28	Organosolv lignin	Ni–Fe/AC	Methanol	200	6	2	20.3	4‐n‐propylsyringol and 4‐n‐propylguaiacol	[75]
29	Birch sawdust						39.5	Phenolic monomer	
30	Rice‐straw lignin	NiMo‐MAC	Formic acid/ethanol	340	6	–	72.2	Lignin oil	[76]
31	Kraft Lignin	20NiMoP/AC	–	400	2	10	45.7	Alkylphenolics, aromatics	[77]
32	Organosolv lignin	W_2_C/AC	Methanol	250	2	0.69	1.7	Phenolic monomer	[78]
33	LA lignin	Ru/ZrO_2_/MWCNT	Methanol	200	2	2.5	11	Phenolic monomer	[79]
34	Enzymatic mild acidolysis lignins	MoOx/CNT	Methanol	260	4	3	38	Meric phenols	[8d]
35	Alkali lignin	N‐STC	Water	250	3	–	7.52	Phenolic monomer	[80]
36	Corn stover	Co/AC‐N	Isopropanol/formic acid/water	235	4	1	23.81	Phenolic monomer	[81]
37	Birch wood	Pd_0.1_/CNx	Methanol	250	3	3	51.4	Lignin‐derived phenolic monomer	[82]
38	Kraft Lignin	ZnO‐Co/N‐CNTs	Water	400	6	5	24.4	Monomers	[83]
39	Cornstalk	Ru/NMC	Ethanol	260	4	1	31.2	Monomer	[84]
40	Birch organosolv lignin	Ru/NCM	Ethanol/water (1:1)	280	2	1	40.7	4‐n‐propylsyringol and 4‐n‐propylguaiacol	[44]
41	Birch organosolv lignin	Ru@NC	Ethanol/water (1:1)	300	2	1	30.5	4‐n‐propylsyringol and 4‐n‐propylguaiacol	[43]
42	Kraft Lignin	Ni‐NDC	Ethanol	150	24	0.8	–	–	[85]
43	Alkali lignin	Ni/CNT	Ethanol	280	0.5	N_2_	66.2	Bio‐oil	[86]
44	Poplar	Ru@NP‐C	Methanol	250	3	2	17.53	Monomer	[87]
45	Sodium lignosulfonate	Ni/NDC	Ethanol/water	250	1	7	17	Monomer	[45]
46	C‐lignin	Ru/ZnO/C	Methanol	200	4	3	67	Catechols	[88]
47	Birch wood	Pd_0.25_/CNx	Methanol	250	3	3	52.7 C %	Phenolic monomer	[47]
48	Biochoice™ Lignin	Ce/CNT	Dioxane/methanol	280	1	4	86.1	Liquefied fuel	[89]
49	Kraft lignin	ZnO/Co@N‐CNTs	Water	350	6	5	–	Lignin‐derived bio‐oil (LBO)	[90]

#### 3.1.1 Pd/AC Catalyst

**Figure 2:**
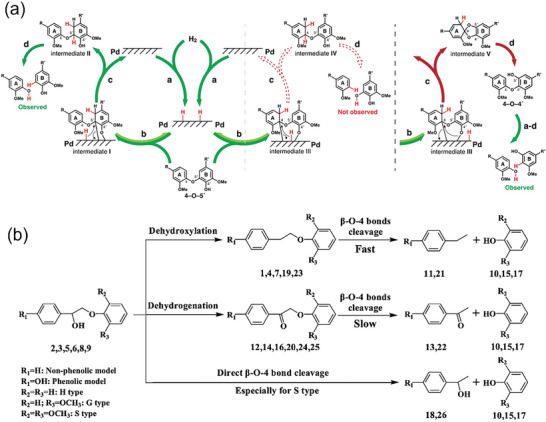
Possible mechanisms of Pd/C‐catalyzed fracture of different lignin C─O bond model compounds. a) 4─O─5 lignin models. Reproduced with permission.^[^ ^28^ ^]^ Copyright 2020, Wiley‐VCH. b) β─O─4 model compounds with different branched chains. Reproduced with permission.^[^ ^31^ ^]^ Copyright 2016, Elsevier.

The heterogeneous catalyst Pd/AC has been extensively studied for its use in lignin hydrogenation.^[^ ^27^ ^]^ The latest research shows that in the hydrogenolysis reaction of diphenyl ethers (DPE), the surface palladium of Pd/AC can adsorb and dissociate H, resulting in the formation of a stable Pd─O bond. This bond facilitates the completion of the elimination reaction, ultimately leading to the production of phenolic monomers.^[^ ^28^ ^]^ The β─O─4 bond compounds without phenyl alcohol are directly and rapidly cleaved to form two aromatic monomers (**Figure** **2**a). However, the dehydrogenation of benzyl alcohol during the cleavage process of the model molecule compound bound to phenyl alcohol hinders the breakage of β─O─4. The key role of phenyl hydrogen atom in breakage of β─O─4 is forming intermediate β‐phenoxyalkylpalladium hydride.^[^ ^29^ ^]^ By introducing a specific quantity of alcohols as a catalytic hydrogen source, it is possible to facilitate the catalyst progression of catalyst through the reaction with a low energy barrier. This reaction follows the mechanism of the transfer hydrogenolysis reaction, that is, the first dehydrogenation, then the adsorption of the enol to Pd/AC, and then the breakage of C─O bond. ^[^ ^30^ ^]^ Moreover, the phenyl carbon‐oxygen bond exhibits a faster cleavage rate compared to the terminal carbon‐oxygen bond, which can only be broken when adjacent phenols are present (Figure 2b).^[^ ^31^ ^]^ Although the research on cracked lignin model compounds has been in‐depth, and the reaction mechanism has become increasingly clear, the cracking mechanism of natural lignin is still not clear enough.

#### 3.1.2 Ru/AC Catalyst

**Figure 3:**
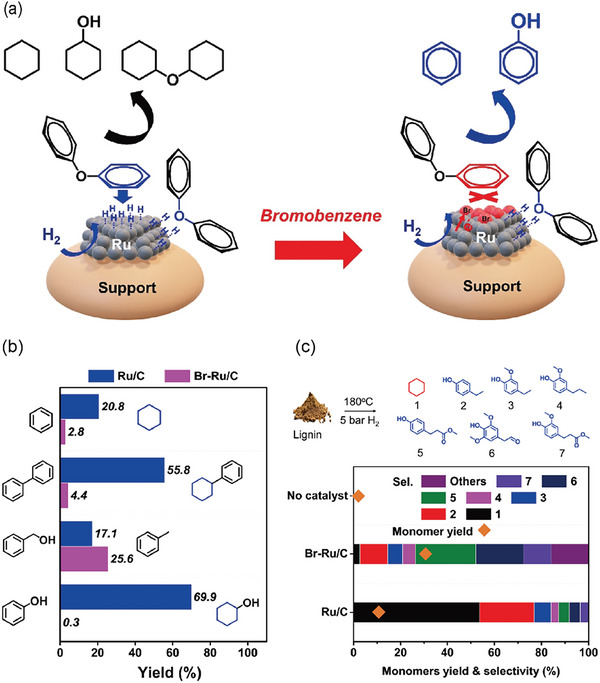
a) Adsorption of diphenyl ethers on catalysts. b) Reactions of different model compounds over catalysts c) Hydrogenolysis of lignin. Reproduced with permission.^[^ ^35c^ ^]^ Copyright 2021, Wiley‐VCH.

Ru/AC is a hydrogenation catalyst with excellent performance, especially for the hydrogenation of lignin and model compounds. When Ru/AC catalyzes the hydrogenolysis of lignin or lignocellulose feedstocks to prepare monomers, the selectivity of the monomers is usually specific. B. F. Sels et al.^[^ ^32^ ^]^ discovered that Ru/AC catalysts exhibit a strong preference for forming para‐propylphenols, resulting in a combined selectivity of 75% towards 4‐n‐propylguaiacol (PG) and 4‐n‐propylsyringol (PS) within the monomer fraction. On the other hand, the presence of Pd/AC catalysts promotes the formation of para‐propanol phenolics, with an impressive selectivity of 91% toward 4‐n‐propanolguaiacol (POHG) and 4‐n‐propanolsyringol (POHS). This difference is attributed to the lower C‐O hydrogenolysis activity of Pd/AC. When compared to other noble metal catalysts, the ratio of propanol‐substituted monomers to propyl‐substituted monomers in phenolic monomers decreases in the following order: Pd > Ru > Pt ≈ Rh.^[^ ^33^ ^]^ When catalyzing the depolymerization of organosolv lignin, soda lignin, and acid hydrolysis lignin, Ru/AC will form less coke than other noble metal catalysts and improve the yield of lignin oil.^[^ ^34^ ^]^ However, Ru/AC shows excessive hydrogenation of phenol. The reason is that hydrogenolysis and hydrogenation reactions happen at the same time, but they occur at different active sites during the catalytic conversion of diphenyl ether. When it comes to hydrogenolysis of C─O bonds, it typically takes place at edge and corner sites,^[^ ^35^ ^]^ whereas hydrogenation of aromatic rings proceeds at successive sites on the platform.^[^ ^36^ ^]^ Selective poisoning of different sites can modulate the distribution of the products. Vitaly V. Ordomsky et al.^[^ ^35c^ ^]^ proposed a heterogeneous Ru/AC catalyst that is modified with Br atoms. The addition of Br selectively inhibits the hydrogenation of aromatic rings by poisoning the platform sites on Ru nanoparticles (**Figure** **3**). However, the edge and corner defect sites remain available and demonstrated higher intrinsic activity in the cleavage of C─O bonds. This modification allows for the selective and direct breakage of the C─O bond in diphenyl ether without any hydrogenation of aromatic rings. The result is a yield of benzene and phenol reaching as high as 90.3% (with almost 0% selectivity on Ru/C).

#### 3.1.3 Other Catalysts

Apart from noble metals, inexpensive and abundant transition metals like Fe,^[^ ^37^ ^]^ Mo,^[^ ^38^ ^]^ Co,^[^ ^39^ ^]^ Cu, and particularly Ni,^[^ ^40^ ^]^ can be used to replace noble metals in the hydrogenation of lignin and lignin‐like model compounds.^[^ ^41^ ^]^ Ni‐based catalysts are not only active in breaking C─O bonds, but also prevent hydrogenation of aromatic hydrocarbons. Benzyl phenyl ether can be catalyzed by Ni/AC to obtain toluene and phenol with high selectivity in 88.5% and 86.5% yields, respectively.^[^ ^42^ ^]^ Ni (0) activates and facilitates the cleavage of C─O bonds in BPE, resulting in the formation of benzyl (C_6_H_5_‐C·) and phenoxy (C_6_H_5_O·) radicals. These radicals then react with the hydrogen atoms released from the reactive surface. Ni/AC is a species that can be used to produce toluene and phenol. It exhibits strong depolymerization activity, breaking both β─O─4 and α─O─4 bonds. John F. Hartwig et al.^[^ ^40a^ ^]^ synthesized Ni/AC with a more vital anti‐sintering ability by combining (Ni(COD))_2_ and activated carbon. The catalyst can produce aromatics and phenols in high yields from di‐ortho‐substituted diaryl ethers. This stability allows cleavage of C─O bonds in highly substituted diaryl ether units, similar to cleavage in lignin. Not only is Ni/C highly active and selective in converting model compounds of lignin, but also efficient in transforming natural lignin into monomeric phenols. The main concept of lignin conversion using the Ni/C catalyst involves breaking down lignin into smaller fragments with molecular weights ranging from 1100 to 1600 through alcoholysis reactions, and then the smaller fragments are double disproportionated on Ni/C catalyst dehydration, and the hydrogenation process of C_α_ and C_β_ and the C_γ_ hydrogenolysis reaction are converted to monomeric phenol.

### 3.2 Nitrogen‐Doped Carbon Supported Catalysts

Nitrogen‐doped carbon (NC) has expansive specific surface area, superb electronic conductivity, and adaptable structure. It has been considered attractive catalyst support. Further, the robust interaction between the nitrogen atoms and metal enhances the dispersion of the active metal. It has high thermal aggregation and leaching stability, changing the acid‐base properties of the support surface and increasing the catalytic activity of the catalyst, making M‐N‐C nanomaterials promising for various catalysis. They have many applications in the hydrogenolysis of lignocellulose/lignin, and exhibit higher catalytic activity than activated carbon‐based catalysts.

**Figure 4:**
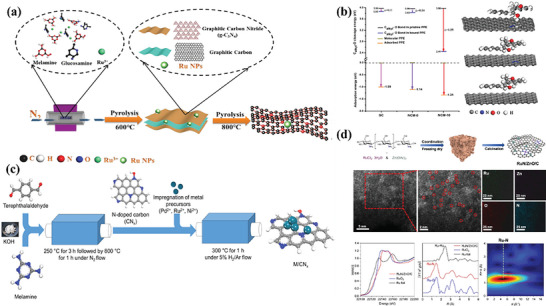
a) Synthesis of M/CNx catalysts. Reproduced with permission.^[^ ^47^ ^]^ Copyright 2020, American Chemical Society. b) Calculation of PPE on catalyst surface. Reproduced with permission.^[^ ^44^ ^]^ Copyright 2020, Elsevier. c) Synthesis and characterization of Ru@N‐doped carbon catalysts. Reproduced with permission.^[^ ^43^ ^]^ Copyright 2019, American Chemical Society. d) Synthesis and characterization of RuN/ZnO/C catalysts. Reproduced with permission.^[^ ^48^ ^]^ Copyright 2022, Springer Nature.

In general, there is more charge transfer between metal with low coordination and N with strong electronegativity, which changes the distribution of electrons around the metal atom, forming stronger chemical bond between the metal atom and the supports, making the dispersion more stable. On the other hand, the presence of nitrogen doping and the inherent defects in carbon materials create additional anchor points for metal atoms. For example, Li et al.^[^ ^43^ ^]^ conducted a two‐stage pyrolysis process to prepare the Ru@N‐C catalyst. This involved using a mixture of d‐glucosamine hydrochloride (GAH) and ruthenium trichloride, with melamine acting as a soft template (depicted in **Figure** **4**a). The thermal condensation of melamine (as a nitrogen precursor) and GAH (as a carbon precursor) resulted in the formation of a carbon skeleton and g‐C_3_N_4_ sandwich structure. This structure effectively hinders the growth of Ru NPs and led to the development of well‐dispersed ultrafine Ru NPs. Additionally, the thermal decomposition of carbon nitride at 800 °C led to the formation of a wrinkled and defect‐rich mesoporous carbon structure, which greatly enhanced the specific surface area. The presence of Ru‐Nx chemical bonds also facilitates the creation of Ru nanoparticles that are highly dispersed. This leads to the ultra‐high activity of this catalyst in lignin hydrogenolysis, with the highest yield of monomers reaching 30.5% at 300 °C. But the yield of Ru@C synthesized by the same method is only 16 wt.%. Based on this work, Li et al.^[^ ^44^ ^]^ synthesized Ru/NCM catalysts by preparing nitrogen‐doped carbon supports and then supporting metal elements. The presence of pyridine N can not only stably disperse Ru NPs as metal coordination but also increase the ratio of Ru^0^. Pyridine N has strong adsorption on 2‐phenoxy‐1‐phenethanol molecules, which reduces the dissociation energy of the C─O bonds (Figure 4b).

The chemical properties of metal can be altered by depositing metallic nickel on NC compared to pristine carbon supports, N acts as an electron donor and reduces the work function of NC supports. The bonding between nickel and NC supports is enhanced through partial electron transfer, leading to an increase in the number of chemically active sites. Molinari et al.^[^ ^45^ ^]^ prepared nitrogen‐doped carbon‐supported Ni nanoparticles with hierarchical porosity by salt melt synthesis. In catalytic sulfate lignin depolymerization, Ni‐NDC is superior in terms of efficiency compared to Ni particles deposited on nitrogen‐free carbon (Ni─C with comparable pore characteristics) as well as Ni nanoparticles deposited on commercial active carbon. Samart et al.^[^ ^46^ ^]^ prepared Ni_2_P/AC‐N catalysts by wet co‐impregnation and H_2_ reduction. The activity and the demethylation of phenolic monomers are greatly enhanced by the good distribution and small crystallites of Ni_2_P, which are achieved as a result of the presence of nitrogen‐containing functional groups on the surface of activated carbon.

Due to the significant presence of carbon and nitrogen in the organic ligands of various MOFs, they serve as suitable sources for generating nitrogen‐doped carbon without requiring an additional nitrogen source.^[^ ^49^ ^]^ The thermal decomposition of MOFs is an essential process for producing metal‐supported nitrogen‐doped carbon materials.^[^ ^50^ ^]^ Unlike bulk materials, MOF materials can endure high‐temperature thermal decomposition without causing significant harm to their structure or appearance.^[^ ^51^ ^]^ Wei et al.^[^ ^52^ ^]^ synthesized Co/C@N catalyst by direct high‐temperature pyrolysis MOF‐ZIF‐67. During the pyrolysis, Co atoms are in situ reduced to Co^0^, and the nitrogen‐containing ligand (2‐methylimidazole) enabled successful nitrogen doping. Benzyl phenyl ether is completely converted with the optimized reaction conditions, and the monomer selectivity reachs 98.2%.

The N heteroatoms in the support can promote the formation of smaller metal nanoparticles and even form single metal atoms to improve catalytic activity. Kim et al.^[^ ^47^ ^]^ prepared CN_x_ supports by calcining melamine and terephthalaldehyde and then prepared Pd/CN catalysts using the impregnation method, resulting in significantly low Pd metal loading (0.25%) (Figure 4c). The CN_x_ supports effectively immobilized the pyridine and pyrrole species within the Pd nanoclusters. Most of Pd in catalyst exists in form of single Pd atom, which significantly improves the activity of the catalyst. the yield of monophenols is 52.5C% from birch (the yield of Pd_0.25_/AC is only 36C%.) at 250 °C for 3 h. In addition, the main product is 4‐n‐propylguaiacol/syringol catalysted by Pd_0.25_/CN_x_, showing unique product selectivity. The RCF performance of Pd_5_/AC and Pd_0.25_/CN_x_ are similar in terms of phenolic monomers yield and degree of delignification results, although the Pd content of Pd_0.25_/CN_x_ is significantly less by a factor of 20 compared to that of Pd_5_/AC. However, the spatio‐temporal yield (measured as the yield of phenolic monomers per Pd loading) of Pd_0.25_/CN_x_ is markedly higher than that of most metal‐loaded catalysts.

A Ru catalyst (RuN/ZnO/C) with low loading and highly dispersion loaded on chitosan‐derived NC was synthesized and used for RCF of lignocellulose by song et al.^[^ ^48^ ^]^ (Figure 4d). This catalyst shows a theoretical maximum yield of phenolic monomers from lignin while preserving cellulose and hemicellulos. Compared to commercial Ru/C catalysts, the RuN/ZnO/C catalyst has a higher atomic economy (twenty times TON) and selectivity of Ru, mainly due to the molecular size of Ru. Furthermore, the catalyst demonstrated excellent stability in cyclic tests and hydrothermal processes, which can be attributed to the coordination of Ru─N species. However, monatomic active site clustering is found in cycling experiments.

The ability catalysts to precipitate hydrogen from alcohols determines the performance of lignin hydrogenolysis in an alcohol‐solvent hydrogen‐free system. Hu et al.^[^ ^53^ ^]^ found that in the RCF of poplar at 160°C, 0.1% H_2_SO_4_, and no hydrogen, phenolic monomer yield of Co SAs‐N@LC is up to 41.7%, gives almost complete delignification of lignin (99.7%), while the Co cluster catalyst synthesized by the same method gave only 25% yield of monophenols, which is mainly attributed to the excellent methanolic hydrogen precipitation ability of monatomic cobalt, which inhibits by reduction the intermediate recondensation by reduction. The DFT calculations revealed that the methanol adsorption energy on the Co SAs‐N@LC surface (−0.636 eV) is slightly higher than on the Co cluster catalyst surface (−0.837 eV); however, both surfaces demonstrate stable methanol adsorption. Comparatively, the Gibbs free energy of H adsorption on the Co SAs‐N@LC surface (0.57 eV) is lower than on the Co cluster catalyst surface (1.38 eV), indicating that Co SAs‐N@LC facilitates the release of adsorbed H, suggesting better hydrogen precipitation performance.

Although some preliminary applications of monoatomic catalysts in lignin hydrogenolysis have been made, more in‐depth studies are necessary to overcome the following problems: on the one hand, how to improve the adsorption of lignin macromolecules by monoatomic catalysts, and on the other hand, how to improve the stability of monoatomic catalysts in lignin hydrogenolysis system and prevent the agglomeration of monoatomic active sites.

## 4 Metal Oxides Supported Catalysts

### 4.1 Monometallic Oxide Supported Catalysts

Metal oxides play an important role in field of catalysis and are widely used as main catalysts, co‐catalysts, and supports. Due to different physic‐chemical properties, different metal oxides exhibit different characteristics in lignin depolymerization, which can regulate the activity and control selectivity of products in lignin depolymerization. **Table** **2** summarizes the recent studies in the hydrogenolysis of lignin or lignocellulose over metal oxides supported catalysts.

#### 4.1.1 Alumina Supported Catalysts

Alumina possesses increased overall acidity, especially when it comes to relevant weak acid sites. These sites can enhance the occurrence of chain scission and ring‐opening reactions.^[^ ^91^ ^]^ Moreover, alumina has a mesoporous structure that aids in the transportation of larger lignin molecules. Therefore, catalysts based on alumina and noble metals typically exhibit superior performance compared to catalysts supported by activated carbon.^[^ ^92^ ^]^ Metal‐free alumina can catalyze the conversion of lignin into lignin‐oil due to its considerable Lewis acidity. However, the repolymerization of the monomers produces a large amount of coke at high temperatures. Pt/Al_2_O_3_ is a well‐known efficient catalyst for hydrogen production from methanol reforming. It can use alcohols as hydrogen source to provide relatively low H* to inhibit excessive hydrogenation of aromatic products.^[^ ^93^ ^]^ For example, Pt/γ‐Al_2_O_3_ can catalyze the hydrogenolysis of model compounds to benzene phenol and benzene with high selectivity (95.7%) in 2‐propanol. But the selectivity to saturated aromatic products was as high as 99% (cyclohexane, cyclohexanol) when used Pt/TiO_2_ catalyst. The Pt/Al_2_O_3_ catalyst exhibits a lower dehydrogenation activity for 2‐propanol compared to the Pt/TiO_2_ catalyst, which inhibits excessive hydrogenation of phenol and benzene.^[^ ^94^ ^]^ Another example, Under optimal conditions (230 °C, 3 MPa N_2_, 3 h),birchwood chips can achieve nearly complete delignification (94%) and yield a significant proportion of high aromatic monomers (49%).^[^ ^95^ ^]^ By adjusting the rate of hydrogenation formation through methanol‐reforming, the selectivity of product can be modified between 4‐propenyl‐syringol and 4‐propyl‐syringol.

#### 4.1.2 Titanium Dioxide Supported Catalysts

Titanium dioxide supports are more easily activated in air/steam than alumina or carbon supports to remove carbon deposits on the catalyst,^[^ ^96^ ^]^ more monomeric aromatic hydrocarbons and alkylphenols can be formed under the synergistic effect with noble metals. Studies of lignin pyrolysis reactions catalyzed by different oxides loaded with Co metals as catalysts showed different product distributions.^[^ ^97^ ^]^ The pyrolysis products are divided into phenols (S, G, and H types), heterocycles, aromatic hydrocarbons, and aliphatic hydrocarbons. Co/CeO_2_ promots the depolymerization of lignin to produce S‐type phenol and shows the greatest selectivity for acetophenone, 1‐(2‐hydroxy‐5‐methylphenyl) and acetosyringone. However, the maximum amount of H‐type phenol (49.7%) is observed in the Co/TiO_2_ catalyst. The effect of Co/TiO_2_ on the formation and degree of demethoxylation of H‐type phenols is more significant than in other catalytic and non‐catalytic systems. In addition, Ofei D.Mante et al.^[^ ^98^ ^]^ studies have shown similar results that titanium dioxide (TiO_2_) selectively removes hydroxyl, carbonyl, and methoxy functional groups from phenolic monomer in lignin, which are primarily used in the production of phenol, cresol, and xylene. The promotion of the deoxygenation reaction is due to the characteristics of the adsorption sites on anatase titanium dioxide and interaction with adsorbed substances. The electronic effects of methoxyl portions andhydroxyl of phenolic monomer cause adsorption (dissociation or molecular) on the titanium dioxide surface. The strong interaction between the surface of anatase and the absorbed substance can break the C─OH (dehydroxylated), C─OCH_3_ (demethoxylated), and O─CH_3_ (demethylated) bonds, resulting in the formation of hydroxyl (OH_ads_), methyl (CH_3ad_), and methoxyl (OCH_3ad_) surface adsorbates, and then hydroxyl group can react to form H_2_O, and the methoxyl group can react to form CH_3_OH, CH_4_S, CH_3_SCH_3_, and even decompose into CO.

#### 4.1.3 Niobium Oxide Supported Catalysts

**Figure 5:**
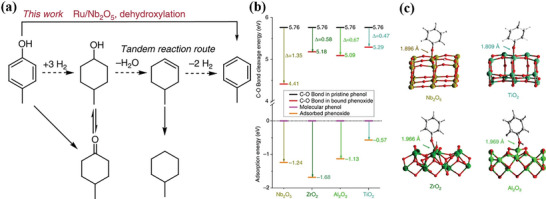
a) Possible conversion pathways for phenol 4‐methylphenol. b) Energy calculations of phenol adsorption on different metal oxides. c) Optimized structural modeling of benzene oxides. Reproduced with permission.^[^ ^100^ ^]^ Copyright 2017, Springer Nature.

Nb_2_O_5_ has become a hot spot in the research on catalytic cracking of C‐O bonds lignin model compounds and lignin due to the water‐resistant Lewis acidity.^[^ ^99^ ^]^ Lignin can be hydrodeoxygenation directly and selectively to produce aromatics in the aqueous phase on porous Ru/Nb_2_O_5_ catalyst (**Table** **3**).^[^ ^100^ ^]^ The yield of birch lignin converted to monomeric C7‐C9 hydrocarbons is close to the theoretical content, the total mass yield of liquid products is 35.5%, and the aromatics selectivity is 71%. The mass yield of C7‐C9 hydrocarbons (18.3 wt.%), phenolic monomers (1.7 wt.%), and aromatic hydrocarbons is (31 wt.%) on Ru/ZrO_2_ catalyst is far lower than Ru/Nb_2_O_5_ (71 wt.%). No C7‐C9 aromatics are detected on Ru/C catalyst, and the lignin monomer is completely yielded by 29.3 wt.% of C7‐C9 naphthenic hydrocarbons. Ru/H‐ZSM‐5 and Ru/TiO_2_ show worse performances for this reaction. Nb_2_O_5_ supports have unique catalytic properties compared to other oxide supports. After being adsorbed on Nb_2_O_5_, the dissociation energy of the C_aromatic_‐OH bond in phenolic substances is significantly reduced (**Figure** **5**b), which makes it have evident selectivity to aromatics. Au/Nb_2_O_5_ catalyst can convert lignin oil into phenolic compounds with high selectivity in aqueous phase and prevent its over‐hydrogenation.^[^ ^101^ ^]^ Compared with Au/ZrO_2,_ Au/TiO_2_ and Au/Al_2_O_3_ catalysts, Au/Nb_2_O_5_ catalysts have higher activity, good stability, and selectivity to phenolic compounds, 2‐methoxy‐4‐propylphenol. The acidic site of the support can catalyze the hydrolysis of 2‐methoxy‐4‐propylphenol to generate propylcatechol, and then effectively dissociate C_aromatic_‐O to generate propylphenol under the synergistic effect of electron‐rich Au and Nb_2_O_5_. In addition, the different forms and morphologies of same supports can also affect the catalytic performance. Wang et al.^[^ ^102^ ^]^ prepared four different morphologies of Nb_2_O_5_ (flowers, hollowed, mesoporous, and layered Nb_2_O_5_) and used them in the HDO study of methyl phenolenol. The variation in specific surface areas of different Nb_2_O_5_ forms gives rise to distinct unsaturated NbO_x_ sites and Ru dispersions, thereby impacting the catalytic activity for hydrodeoxygenation (HDO) and the toluene selectivity of 4‐methylphenol. It has been observed that the Ru catalyst supported on layered structure Nb_2_O_5_ possessed a greater number of Nb═O (unsaturated NbO_x_ sites) and exhibited the highest level of Ru dispersion. As a consequence, this catalyst demonstrated the highest activity and toluene selectivity. When the catalyst is used for lignin conversion, the hydrocarbon yield can reach 99.6%.

**Table 2:** Summary of lignin depolymerization catalyzed by metal oxide‐based catalysts.

	Substrate	Catalyst	Solvent	Temperature [°C]	Time [h]	Pressure [MPa H_2_]	Yield [wt.%]	Main product	Ref
1	Alkali lignin	Ru/Al_2_O_3_	Water	250	4	2	42.98	Monomers	[112]
2	Insoluble lignin	Ru/Al_2_O_3_	Water	250	4	4	21.4	Cyclohexanes	[113]
3	Lignin	Ru/Al_2_O_3_	Formic acid/water	340	6	–	86.8	Lignin oil	[114]
4	Oak lignin	CoMoSx/γ‐Al_2_O_3_	Formic acid/ethanol	340	1	5	13	Lignin bio‐oil	[115]
5	Kraft lignin	Rh/Al_2_O_3_	–	550	4	10	36.3	Lignin oil	[92]
6	Alcell lignin	Ru/TiO_2_	–	400	4	10	78	Lignin oil	[96]
7	Birch lignin	PtRe/TiO_2_	Isopropanol	240	12	He	18.71	Monophenols	[116]
8	Poplar lignin	Pd‐PdO/TiO_2_	Water	180	6	Ar	40	Lignin monomers	[117]
9	Alkali lignin	CaO/CeO_2_	Methanol	180	1	–	53.2	Lignin oil	[118]
10	Locust sawdust	Au_1_Pd_1_/CeO_2_	Ethanol/water	180	5	–	44.1	Monophenols	[119]
11	Birch lignin	Ru/Nb_2_O_5_	Water	250	20	0.7	35.5	Hydrocarbons	[100]
12	Enzymatic lignin	Ru/Nb_2_O_5_	Water	250	20	0.5	99.6	Hydrocarbons	[102]
13	Alkali lignin	5Ni‐5Re/Nb_2_O_5_	Ethanol	330	3	N_2_	35.41	Lignin monomers	[120]
14	Organosolv poplar lignin	Cu_20_PMO	Methanol	180	14	4	54.8	Catechols	[108]
15	Pine	Cu_20_PMO	Methanol	180	18	4	10	Lignin monomers	[13b]
16	Kraft lignin	SA‐Cu/CuMgAlOx	Methanol	360	8	–	93	Lignin oil	[121]
17	Soda lignin	Cu_20_MgAlOx	Ethanol	240	4	N_2_	36	Monomers	[111]
18	Pine sawdust	H‐NiFe_2_O_4_	Methanol/dioxane	250	6	2	15.4	Monomers	[122]
19	Alkaline lignin	Ni/CeO_2_‐ZrO_2_	Water/methanol	250	3h	He	46.8	Lignin oil	[123]

**Table 3:** Hydrodeoxygenation of lignin over different catalysts. Reproduced with permission.^[^ ^100^ ^]^ Copyright 2017, Springer Nature.

Catalyst	Products distribution [wt.%]											Total mass yield [wt.%]
	C7∼C9 arenes			C7∼C9 cycloalkanes			Other C7∼C9	Sum of C7∼C9 Hydrocarbons	C10∼C15	Phenolic monomers	Others	
	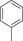	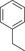	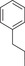	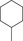	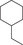	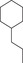						
Ru/Nb_2_O_5_	2.8	9.1	8.5	0.6	4.4	3.6	0.7	29.7	2.2	0.0	3.6	35.5
Ru/ZrO_2_	2.0	3.4	2.1	2.0	5.7	3.1	0.0	18.3	1.5	1.7	2.9	24.4
Ru/A_2_O_3_	0.7	2.7	1.7	0.6	4.4	3.6	0.6	14.3	2.0	2.0	2.4	20.7
Ru/TiO_2_	0.8	1.1	1.0	0.8	1.5	0.9	0.3	6.7	0.2	5.0	2.7	14.6
Ru/HZSM‐5	0.7	0.4	0.7	0.3	0.5	1.7	0.0	4.3	1.5	9.0	1.4	16.2
Ru/C	0.0	0.0	0.0	3.2	14.4	11.7	0.0	29.3	3.7	0.0	0.0	33.0
None	0.0	0.0	0.0	0.0	0.0	0.0	0.0	0.0	0.0	1.3	0.1	1.4
Ru/TiO_2_ Nb_2_O_5_	1.2	1.8	1.1	0.9	2.0	2.0	0.3	9.3	1.1	5.3	2.8	18.5

### 4.2 Mixed Metal Oxides Catalysts

LDHs consist of layers made up of positively charged metal hydroxide and anions in between. Their structure can be considered partially substituted Mg^2+^ cations (Mg(OH)_2_) in brucite and trivalent metal cations. Dispersed in the brucite‐like layer uniformly, Zn^2+^, Ni^2+^, Cu^2+^, Co^2+^ or Fe^3+^, Cr^3+^, and Mn^3+^ cations can be partially replaced by Mg^2+^ or Al^3+^ cations to obtain a variety of LDH materials. Heat‐treating these materials can obtain mixed metal oxides at specific temperatures. These metal oxides have a variety of unique properties, such as high thermal stability, large surface area and porosity, and excellent dispersion of metal oxides, as well as basic properties.^[^ ^103^ ^]^

**Figure 6:**
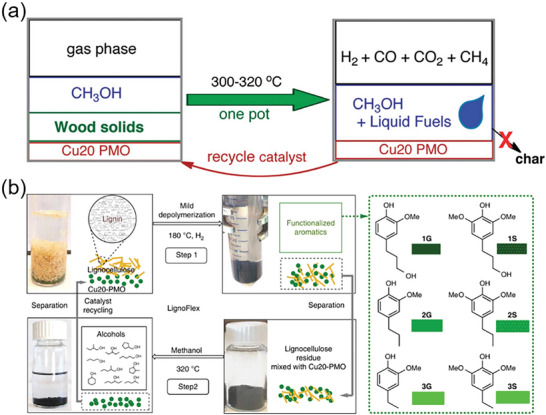
a) Conversion of lignocellulose to fuel. Reproduced with permission.^[^ ^104a^ ^]^ Copyright 2011, American Chemical Society. b) Schematic diagram of the two‐step conversion of lignocellulose. Reproduced with permission.^[^ ^13b^ ^]^ Copyright 2018, Springer Nature.

Mixed metal oxides (MMOs) have been shown to perform hydrolysis and hydrogenation with little or no char formation, improving the efficiency of lignin conversion and helping prevent catalyst inactivation.^[^ ^104^ ^]^ In supercritical methanol, full conversion of lignin can be achieved at relatively mild temperatures (300 °C) using copper‐doped mixed metal oxides as catalysts. When methanol is transferred to the organic solvent lignin, the phenyl ether bonds are fully hydrogenated and the aromatic rings are hydrogenated at the same time. The resulting product is mostly comprised of monomeric substituted cyclohexyl compounds that have lower levels of oxygen and fewer aromatic hydrocarbons.^[^ ^104b^ ^]^ When using lignocellulose as substrate, lignin, cellulose, and hemicellulose can also be fully converted without producing coke. The liquid products are mainly mixtures of C2‐C6 fatty alcohols and their methylated derivatives (**Figure** **6**a).^[^ ^104a^ ^]^ Sun et al.^[^ ^13b^ ^]^ have proposed a catalytic strategy capable of converting lignocellulose into attractive products. The core of strategy is flexible use of Cu_20_PMO in two different stages of lignocellulosic conversion process (Figure 6b). First, lignin oil rich in phenolic monomers is obtained through RCF strategy in the first stage, and then small molecular alcohols are obtained from the remaining national body at high temperature, and comprehensive recovery of the catalyst is achieved through the complete conversion of all process residues.

To further enhance the hydrogenation effect of mixed metal oxides, Huber et al.^[^ ^104^, ^105^ ^]^ reduced mixed metal oxides under a hydrogen atmosphere to obtain Cu/CuMgAlO_x_ and used them for catalytic depolymerization of maple wood, pyrolysis lignin, and maple lignin. The conversion rate of Cu/CuMgAlO_x_ for hydrogenation and deoxygenation of lignin feedstock in supercritical methanol system is higher than Ru/AC, but it still cannot catalyze the break of C─C bonds in lignin.

Although a series of achievements have been achieved in the Cu_20_PMO‐catalyzed lignin depolymerization system in supercritical methanol, one of the key problems is the increase of aromatics hydrogenation and methylation products. These side reactions are mainly due to the high reactivity of the phenolic intermediate.^[^ ^106^ ^]^ High temperature promotes the conversion of lignin and also over‐hydrogenates the benzene ring. At the same time, the pressure of the reaction system increases dramatically and is fraught with danger and instability. Lowering the temperature is beneficial to improve the selectivity of monomers, which is demonstrated in the selectivity of the model compounds Benzyl phenyl ether at lower temperatures.^[^ ^107^ ^]^ The products of natural lignin are mainly phenolic monomer catechol at 140 to 220 °C.^[^ ^108^ ^]^

Control and guidance of the reaction of phenolic intermediates can improve the selectivity of valuable aromatic compounds in mixed oxide catalytic system. For example, dimethyl carbonate triggers the phenol‐intermediate produced by the hydrogenolysis of phenyl ether. As a result, phenol is sequestered to anisole, thereby improving the selectivity of the aromatic product.^[^ ^106^ ^]^ Reactive alkylphenols undergo selective O‐methylation to form alkylmethoxybenzenes, which are less reactive in aromatic hydrogenation. This process helps in preserving aromaticity and decreasing the production of undesirable by‐products.^[^ ^109^ ^]^ In addition, the use of ethanol is more efficient than the use of methanol as a solvent in the Cu_20_PMO catalytic system. The presence of phenolic hydroxyl groups plays a crucial role in repolymerization and the formation of coke. Ethanol can act as a hydrogen‐donating solvent and capping agent to stimulate the stabilization of highly potent intermediates through O‐alkylation of hydroxyl groups and C‐alkylation of aromatic rings.^[^ ^110^ ^]^ Cu_20_PMO catalyst has better performance in lignin hydrogenation compared with Cu/γ‐Al_2_O_3_ and Cu/MgO, Mixed oxides can promote the formaldehyde‐ethanol reaction, thereby inhibiting the polymerization of reactive phenolic with formaldehyde. The copper and base sites of Cu_20_PMO can catalyze the esterification and Gelbert reactions and protect the aldehyde groups of lignin.^[^ ^111^ ^]^ Cu and Al cations (Lewis acid sites) can catalyze O‐ and C‐ alkylation and protect phenolic monomers in lignin oligomers.

## 5 Molecular Sieves Supported Catalysts

Molecular sieves are widely used as solid acid catalysts due to their variable pore structure and tunable Brønsted and Lewis strong acid sites.^[^ ^3^, ^124^ ^]^ The unique topology, large surface area, and morphologies also endear to researchers. Furthermore, molecular sieves exhibit strong adsorption capacity, good hydrothermal stability, electric field properties, and confinement effects in the pore structure.^[^ ^3^, ^124^ ^]^ Therefore, molecular sieves are widely used in industrial practice and laboratory research. The existence of Lewis and Brønsted acid sites in molecular sieves can assist in the enhancement of various biomass‐derived compounds through processes like dealkylation, decarboxylation, dehydration, decarbonylation, isomerization, aromatization, cracking, and dehydrogenation.^[^ ^125^ ^]^ As a result, it has found extensive application as a catalyst or support for breaking down model compounds and lignin. **Table** **4** summarizes the recent studies in the hydrogenolysis of lignin or lignocellulose over molecular sieves supported catalysts.

Molecular sieves usually influence catalytic performances through acidity and pore structure effect. For example, NiCu bimetallic catalysts on different zeolite supports showed better performance in the alcoholysis of lignin. The large surface area with mesopores of support is advantageous for both dispersing the catalyst and converting oxides derived from lignin. NiCu/H‐Beta has the highest oil yield (98.80 wt.%), followed by NiCu/MAS‐7 (95.20 wt.%)> NiCu/MCM‐41 (93.32 wt.%) > NiCu/HZSM‐5 (88.76 wt.%) > NiCu/SAPO‐11 (85.74 wt.%). ^[^ ^126^ ^]^ The pore size of catalyst is positively correlated with the change trend of oil yield. This showed that the larger pore diameter of catalyst, the larger components can be adsorbed and the continuous reaction can be carried out in the lignin depolymerization reaction. The morphology and acidity of molecular sieves significantly affected the product distribution in the molecular sieve‐catalyzed rapid pyrolysis reaction of alkali lignin.^[^ ^127^ ^]^ Y‐molecular sieves is the most efficient catalytic system for the demethoxylation and dehydroxylation of small and large volume oxygenates to aromatics. However, ZSM‐5 and mordenite cannot convert significant oxides due to pore‐clogging and size exclusion. Jelena et al.^[^ ^128^ ^]^ showed similar results in studies of pyrolysis of different species of lignocellulose: The organic phase yield is influenced by both the type of zeolite and its surface area. The decrease in specific surface area and the acidity of the catalysts are associated with the decrease in gas and liquid yields. The structural characteristics of zeolite matrix play important role in the pyrolysis procession. During lignin depolymerization, reactive monomers can be converted to more stable monomers by using molecular sieves with preferred pore sizes. Due to spatial site resistance, the pore system in the molecular sieve hinders the condensation reaction and thus reduces coke formation.^[^ ^129^ ^]^

Compared with other molecular sieves, acid ZSM‐5 has excellent and stable dealkylation performance for 4‐propylphenol, lignin and lignin derivatives to generate phenol.^[^ ^130^ ^]^ The high selectivity of ZSM‐5 due to pore limitation, which avoids disproportionation due to transition state shape selectivity.^[^ ^130a^ ^]^ The competing adsorption of phenol and water reduces the life of phenols at the active site, thus preventing the formation of diphenyl ether in the pores.^[^ ^130b^ ^]^ Nanometer ZSM‐5 has stronger dehydration and deoxidation capacity than micro ZSM‐5.^[^ ^130g^ ^]^ MoOx/SBA15 with oxygen rich defects can induce the adsorption of phenols in a non‐planar manner, weaken the strength of the C_AR_─O_R_ bond, and prevent the aromatics of the ring from excessive hydrogenation.^[^ ^131^ ^]^

**Figure 7:**
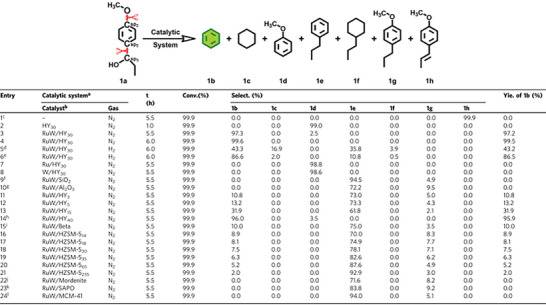
Conversion of 1‐(4‐methoxyphenyl)−1‐propanol over different catalysts. Reproduced with permission.^[^ ^140^ ^]^ Copyright 2021, Springer Nature.

In addition to the pore structure of molecular sieve, acidity is another major factor affecting its activity. Han et al. studied the support effect of RuW alloy catalysts supported on different molecular sieves on the reaction of 1‐(4‐methoxyphenyl)−1‐propanol to benzene (**Figure** **7**). Under the catalysis of Ru/HY_30_, the yield of benzene was almost 100%. Although the conversion of RuW/Beta and RuW/HZSM‐5 to the substrate can also reach nearly 100%, the selectivity of benzene is not satisfactory. HY zeolite with different Si/Al ratio has different acidity, which also affects the selectivity of products. RuW/HY_40_ has appropriate structural properties, which provides high selectivity for benzene. However, it requires 1.5 times the amount of RuW/HY_30_ catalyst due to its low number of Brønsted acid. The acidity generated by the type of zeolite and the ratio of silica to aluminum will affect the reactivity. Another example, in the depolymerization of birch flour, the acidic catalyst 5 wt.% Fe‐beta‐150 promoted the formation of isoeugenol. At the same time, intermediate products such as sinapyl alcohol, coniferyl alcohol, 4‐propenylsyringol, syringaresinol, and syringaldehyde will respond quickly. When used the non‐acid catalyst (5 wt.% Fe‐SiO_2_ and no catalyst), the main products are sinapicate and coniferyl alcohol, respectively.^[^ ^132^ ^]^ According to research, the increase of acid sites can significantly affect the C─O bond breaking, to further increase the monomer yield.^[^ ^133^ ^]^ Lewis acid is the main factor to promotes the pyrolysis of lignin, which directly affects the formation of BTX.^[^ ^134^ ^]^

The dealumination or aluminum addition modification of molecular sieve can adjust the pore structure and acid property, thus further affecting the reaction activity. Li et al.^[^ ^135^ ^]^ synthesized Ni‐supported mesoporous dealuminated β molecular sieve catalyst by oxalic acid dealumination and ammonia water pore expansion. Compared with non‐dealuminated molecular sieves, Ni/DeAl‐beta has a larger pore volume and specific surface area, and the acid sites are decreased, but the total active sites are increased. When used in sulfate lignin liquefaction reactions, the liquid product yield mainly composed of monomers and dimers reached to 88.6%, and the heat of combustion increased to 32.0 MJ kg^−1^.

Due to the ordered pore structure and large specific surface area of mesoporous molecular sieves,^[^ ^136^ ^]^ it is expected to inhibit repolymerization,^[^ ^137^ ^]^ which shows that mesoporous molecular sieve supports are useful for great potential for catalytic upgrading of lignocellulose. SBA‐15 has large pore size and orderly pore structure, which can effectively inhibit repolymerization and reduce coke formation. Adding Ni and Al to SBA‐15 can improve the depolymerization performance of lignin and unstable intermediates.^[^ ^138^ ^]^ On Ni/Al‐SBA‐15(20) catalyst, hydrolyzed lignin is depolymerized in ethanol solvent at 300 °C for 4 h, the liquefaction degree was 81.4%, the monomer yield is 21.90 wt.%, and no evident semi‐coke phenomenon is observed. Adding Ni and Al to SBA‐15 can also improve the acid strength of the catalyst, and the highest bio‐oil yield is obtained in liquefaction reaction of rice straw biomass.^[^ ^139^ ^]^ The Al‐SBA‐15 catalyst was superior to SBA‐15 in terms of phenolic monomer yield and lower coke yield. Al can increase the acidity and activity of SBA‐15 further promoting the splitting of the major bonds of lignin into monomers. But too much aluminum resulting the strong acidity of the catalyst significantly promotes the repolymerization of phenol dimers and oligomers. When Ni is introduced, the degree of liquefaction increases significantly with decreasing focality.

**Table 4:** Summary of lignin depolymerization catalyzed by molecular sieves‐based catalysts.

	Substrate	Catalyst	Solvent	Temperature [°C]	Time [h]	Pressure [MPa H_2_]	Yield [wt.%]	Main product	Ref
1	Kraft lignin	Co‐Zn/Off‐Al H‐beta	Dioxane/methanol	320	6	2	15	Monomeric products	[141]
2	Birch wood	NiO/H‐ZSM‐5	–	500	0.25	N_2_	46	Lignin oil	[128]
3	Kraft lignin	Ni‐Cu/H‐Beta	Isopropanol	330	3	–	50.83	Monomer	[126]
4	Kraft lignin	Ni/DeAl‐beta	Dioxane/methanol	300	36	2	64.5	Lignin oil	[135]
5	Corn stover lignin	NiAPO‐5	Methanol	235	3	2	35.7	Monomer	[133]
6	Organosolv lignin	Ni/HBEA	Hexadecane	273	6	2	70	Hydrocarbons	[142]
7	Organosolv lignin	Ru/H‐molecular sieves β	Ethanol	280	1	2	30	Monomer	[143]
8	Soda lignin	ZSM‐5	–	800	20s	–	18	Monomer	[127]
9	Alkali lignin	HY+ZSM‐5	Ethanol	360	1	N_2_	10.34	Hydrocarbon	[144]
10	Organosolv lignin	2.5Ru−10Ni/Al‐HY	Ethanol	280	5	1	20.2	Phenolic monomers	[145]
11	Pine lignin	RuW/HY_30_	Water	240	12	N_2_	19	Benzene	[140]
12	Enzymatic lignin	Ru/SBA‐15	Isopropanol/water	280	8	2	13.91	Phenolic monomer	[146]
13	Hydrolyzed lignin	Ni/Al‐SBA‐15	Ethanol	300	4	1	21.9	Monomer	[138]
14	Rice straw	Ni/Al‐SBA‐15	Ethanol	280	0.25	4	56.2	Lignin oil	[139]
15	Organosolv lignin	Ni/Al‐SBA‐15	Methylcyclohexane	300	8	4	84	Hydrocarbons	[147]
16	Soda lignin	RuxNi1‐x/SBA‐15	Ethanol	350	40min	–	17.4	Monomeric phenols	[148]
17	Organosolv lignin	Ni/Al‐SBA‐15	Decalin	170	0.5	N_2_	21.36	Phenolic monomer	[149]
18	Native birch lignin	Ni_50_Pd_50_/SBA‐15	Isopropanol/water	245	4	N_2_	37.2	Phenolic monomer	[150]
19	Oak wood sawdust	ZSM‐5	Ethanol	300	0.5	He	32.4	Lignin oil	[151]
20	Kraft lignin	Cu/Mo/ZSM‐5	Ethanol/water	220	7		68	Particular phenol	[152]
21	Alkali lignin	HY	Ethanol	360	1	N_2_	31.26	Hydrocarbon	[144]
22	Kraft lignin	Ni/ZSM‐5	Water	200	4		83	Lignin oil	[153]
23	Organosolv lignin	HZSM‐5	Ethanol/water	350	4		6.5	Monomeric product	[130g]
24	Organosolv lignin	HZSM‐5	Ethanol/water	350	6		9.16	Monomeric product	[130d]

## 6 Metal Synergistic Effect

Although monometal‐based catalysts have achieved specific achievements in the field of depolymerization, their conversion and selectivity still need to be improved. Bimetallic catalysts, which are made by combining two different metals, have attracted widespread attention in technically and scientifically because of their better performance compared to monometallic nanoparticles. Bimetallic catalysts of different shapes, sizes and structures can be synthesized by working on organic or inorganic carriers. Compared to monometallic catalysts, the synergistic effect of bimetallic catalysts enables the potential for enhancing both selectivity and yield.

### 6.1 Noble Based Bimetallic Catalyst

Bimetallic catalysts formed by combining noble metals with low‐cost metals can enhance the catalytic performance of lignin hydrogenolysis. This not only reduces the amount of precious metals and the catalyst cost, but also the catalytic activity is greater than that of either single metal catalyst due to the bimetallic synergy.^[^ ^154^ ^]^ For example, Kim et al.^[^ ^148^ ^]^ studied the depolymerization of soda lignin with bimetallic catalysts under supercritical ethanol conditions. When RuNi catalyst is used, the yield of carbon is the lowest, and the yield of lignin oil is higher that Ru or Ni catalyst. The bimetallic catalyst inhibits recondensation and promotes the understanding polymerization. The highest lignin oil yield (77.5 wt.%) and least carbon (3.2 wt.%) are prepared with Ru_0.6_Ni_0.4_. The ability of the Ru_0.6_Ni_0.4_ catalyst to stabilize unstable products during lignin depolymerization is due to its highest hydrogen desorption capacity. The synergistic effect of RuNi elements and the appropriate element proportion make the catalysis reach the best.

The use of bimetallic catalysts provides many possibilities for lignin depolymerization. The introduction of the second metal as accelerator has been proved to maximize the yield of the required products. Han et al.^[^ ^140^ ^]^ developed a nanoalloy catalyst RuW/HY_30_ that can directly convert lignin to benzene (Figure 7). The monometallic Ru/HY_30_ and W/HY_30_ catalysts have the same function as the HY_30_ zeolite and produce only anisole products. Similarly, bimetallic MW/HY_30_ (M═Co, Fe, Ni, Cu, and Ni) catalyze only the breaking of C_sp2_─C_sp3_ bonds but not C_sp2_─O bonds, demonstrating that in order to conduct the SSH reaction of C_sp2_─O─bonds, it is crucial to have both Ru and W present in combination. Although C_sp2_─O─bonds can be hydrogenated on NiW/HY_30_ catalysts in the presence of exogenous hydrogen, they are much less selective for benzene than RuW/HY_30_ catalysts. The Synergistic effect of RuW alloy can utilize active hydrogen extracted from lignin molecules to catalyze the C_sp2_─O bonds hydrogenation.

In the hydrogenolysis of diphenyl ethers, If Pd and W are alloyed, the conversion increases to 100% and the selectivity becomes 100% toluene. The bimetallic catalyst containing Pd‐W increases the conversion of ether, enhances the process of hydrogenolysis, and improves the selectivity towards toluene and phenol. ^[^ ^155^ ^]^ Typically, using tungsten metals leads to a higher tendency of breaking lower energy aryl ether bonds rather than the higher energy C═C bonds in aromatic rings. The coupling of tungsten with other metals promoted the synergistic effect, indicating that the metal tungsten bimetallic catalyst is more favorable than the single metal catalyst for selective bond breaking of aromatic products.^[^ ^14^, ^78^ ^]^ In addition, the alloy catalysts of Au‐Pd have very high C─O bond breaking activity, resulting the increase in magnitude is nearly tenfold compared to using a single metal catalyst.^[^ ^119^ ^]^ Au‐Pd alloy has solid electronic interaction between Pd and Au atoms. The reduction of palladium electron density significantly improves the adsorption capacity of aromatic carbon‐oxygen bonds and dramatically improves the reaction rate. Using formic acid as a solvent, organic soluble lignin can be directly transformed into valuable monophenols at 180 °C for 6 h, and the yield can reach 44.1%.

PdNi bimetallic catalyst shows higher activity than the parent catalyst in lignin depolymerization. On the larger surface of SBA‐15, Pd^0^ and Ni^0^ bimetallic particles with more uniform distribution can provide more contact opportunities for the rich active centers and the substrate. Among various bimetallic catalysts, Pd_50_Ni_50_/SBA‐15 has the highest total single phenol yield of 8.14 wt.% for Cellulolytic enzyme corn stalk lignin depolymerization at 220 °C for 8 h, and the total single phenol yield of Pd_50_Ni_50_/SBA‐15 for Acid‐extracted birch lignin is 18.52 wt.% at 245 °C. ^[^ ^156^ ^]^ Using cheap nickel instead of a small amount of expensive palladium can greatly improve the catalytic performance of lignin hydrogenolysis to convert to phenol, and greatly reduce the cost of catalyst materials.^[^ ^150^ ^]^ Under normal and neutral conditions, the Pd‐Ni/ZrO_2_ bimetallic nanoparticles can selectively break the β─O─4 bonds of lignin using hydrogen as the donor.^[^ ^157^ ^]^ Nickel and palladium alloys have better activity enhancement and lower hydrogenation selectivity than palladium, showing a significant “synergistic effect”. We can see that the alloying of bimetals can effectively enhance the reaction activity, and significantly increase the selectivity and yield of products.

**Figure 8:**
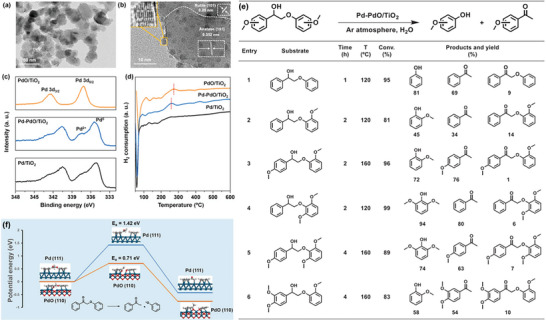
Characterization of a) Pd‐PdO/TiO_2_ catalysts, b) HR‐TEM c) XPS d) H_2_‐TPR e) Substrate expansion for self‐transfer hydrogenation reactions f) DFT calculation of C─O bond cleavage. Reproduced with permission.^[^ ^117^ ^]^ Copyright 2022, Elsevier.

In hydrogenolysis of lignin, not only bimetals can show synergistic effects, but also different states of the same metal can show synergistic effects. Wang et al.^[^ ^117^ ^]^ synthesized Pd/TiO_2_ by impregnation method, and then obtained partially reduced Pd‐PdO/TiO_2_ catalyst by stepwise reduction at room temperature and high temperature (**Figure** **8**). Under the action of this catalyst, a variety of β─O─4 model compounds are cracked into phenolic monomers under hydrogen‐free conditions with high yields. During the reaction process, C_α_H─OH was dehydrogenated on metal palladium to generate β─O─4 ketone intermediate and hydrogen pool, and then β─O─4 ketone intermediate is hydrogenated to decompose the C_β_─O bond through hydrogen pool. Compared with metallic palladium, PdO exhibits a greater efficiency in activating the β─O─4 ketone intermediate and also decreases the activation energy require for cleaving the C─O bond. The synergistic effect of PdO and Pd facilitates the cleavage of β─O─4 chains.

### 6.2 Non‐Noble Based Bimetallic Catalyst

Most of the catalysts combined with nickel and noble metals, such as NiAu^[^ ^158^ ^]^ and NiM (M = Ru, Rh, and Pt), ^[^ ^159^ ^]^ have high depolymerization efficiency for model compounds. However, there are disadvantages, such as over‐hydrogenation of the aromatic ring and low yield of actual lignin conversion, while the combination of transition metals has higher yields. For example, a nickel‐iron alloy catalyst loaded on activated carbon catalyzed birch wood chips RCF with a total monomer yield of 39.5 wt.%. ^[^ ^75^ ^]^ Fe can weaken C─O and promote the hydrogenolysis of C─O in lignin compounds through a synergistic effect. Therefore, the NiFe alloy exhibits a good hydrogenation ability of ether bond in lignin, while preventing excessive hydrogenation of aromatic ring, and the selectivity of PS and PG in the phenolic monomer reaches 88%. Similar experimental results were obtained by Shen et al.^[^ ^160^ ^]^ They suggested that the strong interaction between Ni─Fe, which promotes the reduction of the metal, and the transfer of electrons from Fe to Ni, resulting in the formation of electron‐rich Ni, which can enhance the adsorption of reactive H.

The phenols monomers in lignin oil obtained by the hydrogenation depolymerization of lignocellulose or lignin are usually alkylphenols. However, 4‐propylphenol is more easily hydrogenated to 4‐propylcyclohexanol over single‐metal cobalt‐based catalysts, metals with higher activity are more prone to transition hydrogenation. For example, Lu et al.^[^ ^161^ ^]^ prepared N‐doped bimetallic RuCoNx/NC catalysts by direct pyrolysis of chitin for hydrogenation of model compounds, but the obtained products are mainly over‐hydrogenated. In this case, researchers usually introduce a second weakly reactive metal to tune the selectivity. Nitrogen‐doped carbon catalysts supported by bimetals usually have higher catalytic performances and selectivity due to the synergistic effect between metals. Ma et al.^[^ ^162^ ^]^ synthesized Co_1_‐Fe_0.1_@NC catalysts by pyrolysis of Fe(NO_3_)_3_·9H_2_O and Co(NO_3_)_3_·6H_2_O as precursors and cellulose as a carbon source under ammonia atmosphere. This catalyst can convert lignin in birch and corn stover to 4‐alkylphenol by a two‐step process. When eugenol is used as the model compound, the yield of 4‐propylphenol was 88.3%. Interestingly, the main active phase of the catalyst is CoNx, not the newly formed Co_7_Fe_3_ alloy, and the role of iron is mainly to reduce the adsorption capacity and prevent the further hydrogenation of 4‐alkylphenol from forming 4‐alkylcyclohexanol. These studies have demonstrated the fact that Fe, due to its oxygenophilic nature, not only promotes C─O bond breaking in lignin hydrogenolysis reactions, but also inhibits the hydrogenation of the aromatic ring and obtains a better yield of the monomer.

**Figure 9:**
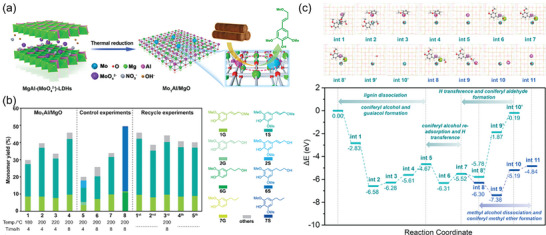
a) Schematic diagram of Mo_1_Al/MgO‐catalyzed lignin conversion b) Lignin depolymerization properties under different conditions. c) DFT calculated energy surface. Reproduced with permission.^[^ ^163^ ^]^ Copyright 2023, American Chemical Society.

Not only the synergism between bimetals, but also the synergism between metal and metal acidic centers can enhance the activity of lignin hydrogenolysis. For example, the addition of zinc increases the catalyst acidity and promotes lignin depolymerization and condensation. However, when Co is added to Off‐Al H‐beta, it causes a decrease in the acidity of the catalyst. As a result, the depolymerization and condensation of lignin also decreased. Both the yield of solid product (11.6%) and the yield of petroleum ether soluble product (30.4%) are lower. This means that the presence of Co, which acts as a hydrogen‐binding site, can effectively decrease the amount of solid product by stabilizing the intermediate of lignin depolymerization. On the other hand, Zn can promote the depolymerization of lignin. By using bimetallic catalysts of Co─Zn/Off‐Al H with different metal loading ratios, it is possible to optimize these opposing reactions. The highest yield of petroleum ether soluble products (54.8%) is achieved when the Co─Zn ratio was 1:3.^[^ ^141^ ^]^ Wang et al.^[^ ^163^ ^]^ have synthesized a dual active site catalyst with a Mo‐O active center and an Al Lewis acidic site on a MgO carrier by LDH‐based anion intercalation and 2D stabilization strategy (**Figure** **9**). Mo_1_Al/MgO has a very high reactivity towards lignin model compounds. The Mo_1_‐O_5_ center and the adjacent Al site have various functions during different stages of the reaction. Initially, both the Mo and Al Lewis acid sites can capture the reaction intermediates of model compound depolymerization. Once guaiacol is formed and released, the Mo site stabilizes the O* intermediate and serves as an oxidant to convert coniferyl alcohol into coniferyl aldehyde and coniferic acid.

## 7 Summary and Outlook

In this review, the application of various catalysts in reductive depolymerization of lignin, lignocellulose and model compounds is systematically introduced to improve the selectivity and yield of target products. In lignin hydrogenolysis reactions, different types of catalysts have different feature: The activity of activated carbon‐based catalysts is mainly regulated by active metals. The phenolic monomers produced by Ru‐based catalysts are mainly propyl phenols, while those produced by Pd based catalysts are mainly propyl alcohol‐based phenols. Nitrogen‐doped carbon supports can make active metals more dispersed and have high stability to thermal aggregation and leaching. Even further disperses the metal into single atoms, further improving atomic utilization. Alumina exhibits greater overall acidity, specifically an increased presence of weak acid sites that contribute to the promotion of chain scission and ring‐opening reactions. Additionally, its mesoporous structure aids in the transportation of larger lignin molecules. Titanium dioxide supports are more easily activated in air/steam than alumina or carbon supports to remove carbon deposits on the catalyst. Nb_2_O_5_ has a water‐resistant Lewis acid that can catalyze lignin and C─O bond aqueous catalytic cracking. Mixed metal oxides (MMOs) of hydrotalcite‐like precursors can undergo hydrolysis and hydrogenolysis with little or no char formation, enhancing the effectiveness of lignin conversion and aiding in the prevention of catalyst deactivation. Using molecular sieves with preferred pore sizes during lignin depolymerization, reactive monomers can be converted to more stable ones. Due to steric hindrance, the pore system in molecular sieves can inhibit bimolecular condensation, improve yield of monomers and reduce coke formation. The bimetallic catalyst formed by combining noble metals with low‐cost metals can enhance the catalytic performance of lignin hydrogenolysis, and reduces the number of precious metals and catalyst costs.

Although heterogeneous catalysts have achieved staged achievements in lignin depolymerization, there are still many challenges to be faced. 1) The catalytic mechanism of hydrogenolysis of lignin or lignocellulose is still not clear enough. At present, it is believed that the essence of lignin hydrogenolysis is that the large molecule lignin is broken into small molecule lignin fragments or active intermediates under the dual action of solvent and catalyst, and then the active intermediates are stabilized as phenolic monomers in the metal or acidic sites of the catalyst. However, due to the highly interconnected macromolecular structure of lignin, the detailed mechanism of real lignin hydrogenolysis is still not clear enough. In the future, we should further develop the new technology of lignin structure analysis, and at the same time, we should develop and synthesize model compounds that can represent the real structure of lignin, and we should further clarify the mechanism of lignin hydrogenolysis by using in situ characterization technology. 2) current catalysts focus on the selective depolymerization of C─O bonds in lignin. New research should focus on C─C bonds with more muscular bond energy. Selective cleavage of bonds to further improve the yield of the product. 3) The last and most difficult challenge is depolymerizing lignin into a single platform compound in high yield and obtaining products with specific structures. While phenol is also an exciting target at the moment, it would be beneficial for future studies to also prioritize the exploration of novel approaches that can enhance the practical applications and value of these developing, more advanced structures in the production of tangible goods. While the challenges for future development remain daunting, there is no doubt that the exciting new developments achieved so far offer a bright future for lignin value addition in the years to come.

## Conflict of Interest

The authors declare no conflict of interest.

## References

[1] S. H. Mohr , J. Wang , G. Ellem , J. Ward , D. Giurco , Fuel 2015, 141, 120.

[2] a) T. Ren , S. You , Z. Zhang , Y. Wang , W. Qi , R. Su , Z. He , Green Chem. 2021, 23, 1648;

[3] a) A. Pineda , A. F. Lee , Appl. Petrochem. Res. 2016, 6, 243; 32355588 10.1007/s13203-016-0157-y PMC7175707

[4] a) J. Ralph , Phytochem. Rev. 2009, 9, 65;

[5] a) Y. Jing , L. Dong , Y. Guo , X. Liu , Y. Wang , ChemSusChem 2020, 13, 4181; 31886600 10.1002/cssc.201903174

[6] a) J. G. Linger , D. R. Vardon , M. T. Guarnieri , E. M. Karp , G. B. Hunsinger , M. A. Franden , C. W. Johnson , G. Chupka , T. J. Strathmann , P. T. Pienkos , G. T. Beckham , Proc. Natl. Acad. Sci. USA 2014, 111, 12013; 25092344 10.1073/pnas.1410657111 PMC4143016

[7] K. Pan , M. Tian , Z.‐H. Jiang , B. Kjartanson , A. Chen , Electrochim. Acta 2012, 60, 147.

[8] a) Y. Jiang , Z. Li , X. Tang , Y. Sun , X. Zeng , S. Liu , L. Lin , Energy Fuels 2015, 29, 1662;

[9] a) K. Stärk , N. Taccardi , A. Bösmann , P. Wasserscheid , ChemSusChem 2010, 3, 719; 20480494 10.1002/cssc.200900242

[10] a) R. Jastrzebski , S. Constant , C. S. Lancefield , N. J. Westwood , B. M. Weckhuysen , P. C. A. Bruijnincx , ChemSusChem 2016, 9, 2074; 27440544 10.1002/cssc.201600683 PMC5129541

[11] a) Z. Sun , B. Fridrich , A. De Santi , S. Elangovan , K. Barta , Chem. Rev. 2018, 118, 614; 29337543 10.1021/acs.chemrev.7b00588 PMC5785760

[12] S. Van Den Bosch , T. Renders , S. Kennis , S.‐F. Koelewijn , G. Van Den Bossche , T. Vangeel , A. Deneyer , D. Depuydt , C. M. Courtin , J. M. Thevelein , W. Schutyser , B. F. Sels , Green Chem. 2017, 19, 3313.

[13] a) Z. Sun , J. Cheng , D. Wang , T.‐Q.i Yuan , G. Song , K. Barta , ChemSusChem 2020, 13, 5199; 32748524 10.1002/cssc.202001085

[14] a) X. Liu , F. P. Bouxin , J. Fan , V. L. Budarin , C. Hu , J. H. Clark , ChemSusChem 2020, 13, 4296; 32662564 10.1002/cssc.202001213 PMC7540457

[15] W. Boerjan , J. Ralph , M. Baucher , Annu. Rev. Plant Biol. 2003, 54, 519. 14503002 10.1146/annurev.arplant.54.031902.134938

[16] J. S. Luterbacher , A. Azarpira , A. H. Motagamwala , F. Lu , J. Ralph , J. A. Dumesic , Energy Environ. Sci. 2015, 8, 2657.

[17] L. Shuai , M. T. Amiri , Y. M. Questell‐Santiago , F. Héroguel , Y. Li , H. Kim , R. Meilan , C. Chapple , J. Ralph , J. S. Luterbacher , Science 2016, 354, 329. 27846566 10.1126/science.aaf7810

[18] a) N. Yan , C. Zhao , P. J. Dyson , C. Wang , L.‐T. Liu , Y. Kou , ChemSusChem 2008, 1, 626; 18702164 10.1002/cssc.200800080

[19] J. Y. Zhu , X. Pan , R. S. Zalesny Jr. , Appl. Microbiol. Biotechnol. 2010, 87, 847. 20473606 10.1007/s00253-010-2654-8

[20] C. Li , X. Zhao , A. Wang , G. W. Huber , T. Zhang , Chem. Rev. 2015, 115, 11559. 26479313 10.1021/acs.chemrev.5b00155

[21] H. Chung , N. R. Washburn , Green Mater. 2013, 1, 137.

[22] T. Wang , S. Tan , C. Liang , Carbon 2009, 47, 1880.

[23] a) D. Angin , Fuel 2014, 115, 804;

[24] A. Veksha , E. Sasaoka , M.d. A. Uddin , Carbon 2009, 47, 2371.

[25] A. Bagreev , J. Angel Menendez , I. Dukhno , Y. Tarasenko , T. J. Bandosz , Carbon 2004, 42, 469.

[26] W. Ao , J. Fu , X. Mao , Q. Kang , C. Ran , Y. Liu , H. Zhang , Z. Gao , J. Li , G. Liu , J. Dai , Renewable Sustainable Energy Rev. 2018, 92, 958.

[27] a) J. A. Onwudili , P. T. Williams , Green Chem. 2014, 16, 4740;

[28] Y. Li , S. D. Karlen , B. Demir , H. Kim , J. Luterbacher , J. A. Dumesic , S. S. Stahl , J. Ralph , ChemSusChem 2020, 13, 4487. 32202385 10.1002/cssc.202000753

[29] X. Zhou , J. Mitra , T. B. Rauchfuss , ChemSusChem 2014, 7, 1623. 24692272 10.1002/cssc.201301253

[30] M. V. Galkin , C. Dahlstrand , J. S. M. Samec , ChemSusChem 2015, 8, 2187. 25925736 10.1002/cssc.201500117

[31] G. Zhu , X. Ouyang , L. Jiang , Y. Zhu , D. Jin , Y. Pang , X. Qiu , Fuel Process. Technol. 2016, 154, 132.

[32] S. Van Den Bosch , W. Schutyser , S.‐F. Koelewijn , T. Renders , C. M. Courtin , B. F. Sels , Chem. Commun. (Camb) 2015, 51, 13158. 26086373 10.1039/c5cc04025f

[33] T. Renders , E. Cooreman , S. Van Den Bosch , W. Schutyser , S.‐F. Koelewijn , T. Vangeel , A. Deneyer , G. Van Den Bossche , C. M. Courtin , B. F. Sels , Green Chem. 2018, 20, 4607.

[34] a) X.‐J. Shen , P.‐L. Huang , J.‐L. Wen , R.‐C. Sun , Fuel Process. Technol. 2017, 167, 491;

[35] a) L. Zhang , M. Zhou , A. Wang , T. Zhang , Chem. Rev. 2020, 120, 683; 31549814 10.1021/acs.chemrev.9b00230

[36] a) C. Mondelli , G. Gözaydin , N. Yan , J. Pérez‐Ramírez , Chem. Soc. Rev. 2020, 49, 3764; 32459227 10.1039/d0cs00130a

[37] D. J. Rensel , S. Rouvimov , M. E. Gin , J. C. Hicks , J. Catal. 2013, 305, 256.

[38] T. Prasomsri , M. Shetty , K. Murugappan , Y. Román‐Leshkov , Energy Environ. Sci. 2014, 7, 2660.

[39] X. Liu , L. Xu , G. Xu , W. Jia , Y. Ma , Y. Zhang , ACS Catal. 2016, 6, 7611.

[40] a) F. Gao , J. D. Webb , J. F. Hartwig , Angew. Chem., Int. Ed. 2016, 55, 1474; 10.1002/anie.201509133 26666391

[41] I. Klein , B. Saha , M. M. Abu‐Omar , Catal. Sci. Technol. 2015, 5, 3242.

[42] C. Zhu , J.‐P. Cao , X.‐Y. Zhao , T. Xie , J. Ren , X.‐Y. Wei , J. Energy Inst. 2019, 92, 74.

[43] T. Li , H. Lin , X. Ouyang , X. Qiu , Z. Wan , ACS Catal. 2019, 9, 5828.

[44] T. Li , H. Lin , X. Ouyang , X. Qiu , Z. Wan , T. Ruan , Fuel 2020, 278, 118324.

[45] F. Brandi , M. Antonietti , M. Al‐Naji , Green Chem. 2021, 23, 9894.

[46] P. Panpian , L. K. H. Pham , S. Kongparakul , M. Ding , P. Wang , G. Guan , N. Chanlek , Y. Poo‐Arporn , P. Reubroycharoen , C. Samart , J. Environ. Chem. Eng. 2021, 9, 106702.

[47] J. Park , H. S. Cahyadi , U. Mushtaq , D. Verma , D. Han , K.‐W. Nam , S. K. Kwak , J. Kim , ACS Catal. 2020, 10, 12487.

[48] Z. Liu , H. Li , X. Gao , X. Guo , S. Wang , Y. Fang , G. Song , Nat. Commun. 2022, 13, 4716. 35953497 10.1038/s41467-022-32451-5 PMC9372153

[49] K. Shen , X. Chen , J. Chen , Y. Li , ACS Catal. 2016, 6, 5887.

[50] a) M. Drack , I. Graz , T. Sekitani , T. Someya , M. Kaltenbrunner , S. Bauer , Adv. Mater. 2015, 27, 34; 25332107 10.1002/adma.201403093 PMC4315904

[51] X. Li , Y. Fang , X. Lin , M. Tian , X. An , Y. Fu , R. Li , J. Jin , J. Ma , J. Mater. Chem. A 2015, 3, 17392.

[52] Q.‐L. Song , Y.‐P. Zhao , F.‐P. Wu , G.‐S. Li , X. Fan , R.‐Y. Wang , J.‐P. Cao , X.‐Y. Wei , Renewable Energy 2020, 148, 729.

[53] J. Ge , G. Wang , W. Sui , C. Si , H. Guo , Y. Ni , J. Hu , Chem. Eng. J. 2023, 462, 142109.

[54] W. Schutyser , S. Van Den Bosch , T. Renders , T. De Boe , S.‐F. Koelewijn , A. Dewaele , T. Ennaert , O. Verkinderen , B. Goderis , C. M. Courtin , B. F. Sels , Green Chem. 2015, 17, 5035.

[55] T. Renders , S. Van Den Bosch , T. Vangeel , T. Ennaert , S.‐F. Koelewijn , G. Van Den Bossche , C. M. Courtin , W. Schutyser , B. F. Sels , ACS Sustainable Chem. Eng. 2016, 4, 6894.

[56] K. Y. Nandiwale , A. M. Danby , A. Ramanathan , R. V. Chaudhari , A. H. Motagamwala , J. A. Dumesic , B. Subramaniam , ACS Sustainable Chem. Eng. 2020, 8, 4096.

[57] F. Gao , J. D. Webb , H. Sorek , D. E. Wemmer , J. F. Hartwig , ACS Catal. 2016, 6, 7385.

[58] R. Shu , Y. Xu , L. Ma , Q. Zhang , C. Wang , Y. Chen , Chem. Eng. J. 2018, 338, 457.

[59] R. Shu , J. Long , Y. Xu , L. Ma , Q. Zhang , T. Wang , C. Wang , Z. Yuan , Q. Wu , Bioresour. Technol. 2016, 200, 14. 26476159 10.1016/j.biortech.2015.09.112

[60] T. Parsell , S. Yohe , J. Degenstein , T. Jarrell , I. Klein , E. Gencer , B. Hewetson , M. Hurt , J. I. Kim , H. Choudhari , B. Saha , R. Meilan , N. Mosier , F. Ribeiro , W. N. Delgass , C. Chapple , H. I. Kenttämaa , R. Agrawal , M. M. Abu‐Omar , Green Chem. 2015, 17, 1492.

[61] T. Renders , W. Schutyser , S. Van Den Bosch , S.‐F. Koelewijn , T. Vangeel , C. M. Courtin , B. F. Sels , ACS Catal. 2016, 6, 2055.

[62] H. Zhang , H. Zhang , S. Tian , S. Fu , Bioresour. Technol. 2021, 341, 125848. 34467890 10.1016/j.biortech.2021.125848

[63] X. Huang , J. Zhu , T. I. Korányi , M. D. Boot , E. J. M. Hensen , ChemSusChem 2016, 9, 3262. 27767255 10.1002/cssc.201601252

[64] W. Yang , X. Li , X. Du , Y. Deng , H. Dai , Catal. Commun. 2019, 126, 30.

[65] W. Lv , Z. Si , Z. Tian , C. Wang , Q. Zhang , Y. Xu , T. Wang , L. Ma , ACS Sustainable Chem. Eng. 2017, 5, 2981.

[66] R. Shu , Y. Xu , L. Ma , Q. Zhang , T. Wang , P. Chen , Q. Wu , RSC Adv. 2016, 6, 88788.

[67] X. Liu , F. P. Bouxin , J. Fan , V. L. Budarin , C. Hu , J. H. Clark , J. Hazard. Mater. 2021, 402, 123490. 32712365 10.1016/j.jhazmat.2020.123490

[68] B. M. Matsagar , Z.‐Y. Wang , C. Sakdaronnarong , S. S. Chen , D. C. W. Tsang , K. C.‐W. Wu , ChemCatChem 2019, 11, 4604.

[69] D. G. Brandner , J. S. Kruger , N. E. Thornburg , G. G. Facas , J. K. Kenny , R. J. Dreiling , A. R. C. Morais , T. Renders , N. S. Cleveland , R. M. Happs , R. Katahira , T. B. Vinzant , D. G. Wilcox , Y. Román‐Leshkov , G. T. Beckham , Green Chem. 2021, 23, 5437.

[70] H. Luo , I. M. Klein , Y. Jiang , H. Zhu , B. Liu , H. I. Kenttämaa , M. M. Abu‐Omar , ACS Sustainable Chem. Eng. 2016, 4, 2316.

[71] M. L. Stone , E. M. Anderson , K. M. Meek , M. Reed , R. Katahira , F. Chen , R. A. Dixon , G. T. Beckham , Y. Román‐Leshkov , ACS Sustainable Chem. Eng. 2018, 6, 11211.

[72] J. Park , A. Riaz , D. Verma , H. J. Lee , H. M. Woo , J. Kim , ChemSusChem 2019, 12, 1743. 30702216 10.1002/cssc.201802847

[73] B. Du , C. Liu , X. Wang , Y. Han , Y. Guo , H. Li , J. Zhou , Renewable Energy 2020, 147, 1331.

[74] C. Cheng , P. Li , W. Yu , D. Shen , S. Gu , Bioresour. Technol. 2021, 319, 124238. 33254461 10.1016/j.biortech.2020.124238

[75] Y. Zhai , C. Li , G. Xu , Y. Ma , X. Liu , Y. Zhang , Green Chem. 2017, 19, 1895.

[76] M. Oregui‐Bengoechea , N. Miletic , W. Hao , F. Björnerbäck , M. H. Rosnes , J. S. Garitaonandia , N. Hedin , P. L. Arias , T. Barth , ACS Sustainable Chem. Eng. 2017, 5, 11226.

[77] R. K. Chowdari , S. Agarwal , H. J. Heeres , ACS Sustainable Chem. Eng. 2019, 7, 2044. 30775190 10.1021/acssuschemeng.8b04411 PMC6369682

[78] H. Guo , B. Zhang , Z. Qi , C. Li , J. Ji , T. Dai , A. Wang , T. Zhang , ChemSusChem 2017, 10, 523. 27863130 10.1002/cssc.201601326

[79] B. Gómez‐Monedero , M. Pilar Ruiz , F. Bimbela , J. Faria , Fuel Process. Technol. 2018, 173, 165.

[80] S. Totong , P. Daorattanachai , A. T. Quitain , T. Kida , N. Laosiripojana , Ind. Eng. Chem. Res. 2019, 58, 13041.

[81] C. Shen , W. Li , B. Zhang , F. Xue , X. Dou , X. Zhang , Y. Jiang , Mol. Catal. 2021, 514, 111832.

[82] J. Park , U. Mushtaq , J. R. Sugiarto , D. Verma , J. Kim , Appl. Catal. B 2022, 310, 121280.

[83] U. Mushtaq , J. Park , A. Riaz , V. Ranaware , M. K. Khan , D. Verma , J. Kim , ACS Sustainable Chem. Eng. 2021, 9, 3232.

[84] B. Luo , L. Zhou , Z. Tian , Y. He , R. Shu , Fuel Process. Technol. 2022, 231, 107218.

[85] S. M. G. Lama , J. Pampel , T.‐P. Fellinger , V. P. Beskoski , L. Slavkovic‐Beskoski , M. Antonietti , V. Molinari , ACS Sustainable Chem. Eng. 2017, 5, 2415.

[86] P. Jia , J. Wang , W. Zhang , J. Energy Inst. 2021, 94, 1.

[87] T. Ding , Y. Wu , X. Zhu , G. Lin , X. Hu , H. Sun , Y. Huang , S. Zhang , H. Zhang , ACS Sustainable Chem. Eng. 2022, 10, 2343.

[88] S. Wang , K. Zhang , H. Li , L.‐P. Xiao , G. Song , Nat. Commun. 2021, 12, 416. 33462206 10.1038/s41467-020-20684-1 PMC7814062

[89] Q. Ma , Q. Liu , W. Li , L. Ma , J. Wang , M. Liu , Q. Zhang , Fuel Process. Technol. 2017, 161, 220.

[90] V. Ranaware , D. Verma , R. Insyani , A. Riaz , S. M. Kim , J. Kim , Green Chem. 2019, 21, 1021.

[91] M. Oregui Bengoechea , A. Hertzberg , N. Miletic , P. L. Arias , T. Barth , J. Anal. Appl. Pyrolysis 2015, 113, 713.

[92] I. Hita , P. J. Deuss , G. Bonura , F. Frusteri , H. J. Heeres , Fuel Process. Technol. 2018, 179, 143.

[93] a) Z. Q. Bo Zhang , X. Li , J. Ji , L. Zhang , H. Wang , X. Liu , C. Li , Green Chem. 2019, 21, 5556;

[94] C. Zhu , S. Ding , H. Hojo , H. Einaga , ACS Catal. 2021, 11, 12661.

[95] X. Ouyang , X. Huang , J. Zhu , M. D. Boot , E. J. M. Hensen , ACS Sustainable Chem. Eng. 2019, 7, 13764.

[96] A. Kloekhorst , H. J. Heeres , ACS Sustainable Chem. Eng. 2015, 3, 1905.

[97] A. Kumar , B. Biswas , K. Saini , A. Kumar , J. Kumar , B. B. Krishna , T. Bhaskar , Renewable Energy 2021, 172, 121.

[98] O. D. Mante , J. A. Rodriguez , S. P. Babu , Bioresour. Technol. 2013, 148, 508. 24080289 10.1016/j.biortech.2013.09.003

[99] a) G. F. Leal , S. Lima , I. Graça , H. Carrer , D. H. Barrett , E. Teixeira‐Neto , A. A. S. Curvelo , C. B. Rodella , R. Rinaldi , iScience 2019, 15, 467; 31125909 10.1016/j.isci.2019.05.007 PMC6532020

[100] Y. Shao , Q. Xia , L. Dong , X. Liu , X. Han , S. F. Parker , Y. Cheng , L. L. Daemen , A. J. Ramirez‐Cuesta , S. Yang , Y. Wang , Nat. Commun. 2017, 8, 16104. 28737172 10.1038/ncomms16104 PMC5527281

[101] L. Dong , Y. Xin , X. Liu , Y. Guo , C.‐W. Pao , J.‐L. Chen , Y. Wang , Green Chem. 2019, 21, 3081.

[102] Y. Xin , L. Dong , Y. Guo , X. Liu , Y. Hu , Y. Wang , J. Catal. 2019, 375, 202.

[103] a) T. Baskaran , J. Christopher , A. Sakthivel , RSC Adv. 2015, 5, 98853;

[104] a) T. D. Matson , K. Barta , A. V. Iretskii , P. C. Ford , J. Am. Chem. Soc. 2011, 133, 14090; 21806029 10.1021/ja205436c

[105] J. Li , P. H. Galebach , J. K. Johnson , T. Fredriksen , A. Wittrig , X. Bai , H. Yang , G. W. Huber , Green Chem. 2020, 22, 8403.

[106] C. M. Bernt , G. Bottari , J. A. Barrett , S. L. Scott , K. Barta , P. C. Ford , Catal. Sci. Technol. 2016, 6, 2984.

[107] X. Kong , C. Liu , M. Lei , F. Gong , Y. Han , Y. Fan , M. Li , R. Xiao , ACS Sustainable Chem. Eng. 2021, 9, 10939.

[108] K. Barta , G. R. Warner , E. S. Beach , P. T. Anastas , Green Chem. 2014, 16, 191.

[109] J. A. Barrett , Y. Gao , C. M. Bernt , M. Chui , A. T. Tran , M. B. Foston , P. C. Ford , ACS Sustainable Chem. Eng. 2016, 4, 6877.

[110] X. Huang , T. I. Korányi , M. D. Boot , E. J. M. Hensen , ChemSusChem 2014, 7, 2276. 24867490 10.1002/cssc.201402094

[111] X. Huang , C. Atay , T. I. Korányi , M. D. Boot , E. J. M. Hensen , ACS Catal. 2015, 5, 7359.

[112] S. Chen , Q. Lu , W. Han , P. Yan , H. Wang , W. Zhu , Fuel 2021, 283, 119333.

[113] H. Wang , H. Ben , H. Ruan , L. Zhang , Y. Pu , M. Feng , A. J. Ragauskas , B. Yang , ACS Sustainable Chem. Eng. 2017, 5, 1824.

[114] M. Oregui Bengoechea , N. Miletíc , M. H. Vogt , P. L. Arias , T. Barth , Bioresour. Technol. 2017, 234, 86. 28319777 10.1016/j.biortech.2017.02.129

[115] M. J. Hidajat , A. Riaz , J. Kim , Chem. Eng. J. 2018, 348, 799.

[116] J. Hu , S. Zhang , R. Xiao , X. Jiang , Y. Wang , Y. Sun , P. Lu , Bioresour. Technol. 2019, 279, 228. 30735932 10.1016/j.biortech.2019.01.132

[117] Z. Dou , Z. Zhang , M. Wang , Appl. Catal. B 2022, 301, 120767.

[118] B. Biswas , A. Kumar , B. B. Krishna , T. Bhaskar , Renewable Energy 2021, 175, 270.

[119] X. Gao , S. Zhu , Y. Li , Mol. Catal. 2019, 462, 69.

[120] L. Kong , L. Zhang , J. Gu , L. Gou , L. Xie , Y. Wang , L. Dai , Bioresour. Technol. 2020, 299, 122582. 31877480 10.1016/j.biortech.2019.122582

[121] X. Kong , C. Liu , Y. Fan , W. Xu , R. Xiao , ACS Sustainable Chem. Eng. 2022, 10, 495.

[122] X. Dou , W. Li , C. Zhu , X. Jiang , Appl. Catal. B 2021, 287, 119975.

[123] S. Totong , P. Daorattanachai , N. Laosiripojana , R. Idem , Fuel Process. Technol. 2020, 198, 106248.

[124] X. Li , G. Chen , C. Liu , W. Ma , B. Yan , J. Zhang , Renewable Sustainable Energy Rev. 2017, 71, 296.

[125] a) L. Fan , Y. Zhang , S. Liu , N. Zhou , P. Chen , Y. Cheng , M. Addy , Q. Lu , M. M. Omar , Y. Liu , Y. Wang , L. Dai , E. Anderson , P. Peng , H. Lei , R. Ruan , Bioresour. Technol. 2017, 241, 1118; 28578807 10.1016/j.biortech.2017.05.129

[126] L. Kong , C. Liu , J.i Gao , Y. Wang , L. Dai , Bioresour. Technol. 2019, 276, 310. 30641329 10.1016/j.biortech.2019.01.015

[127] A. Kumar , A. Kumar , J. Kumar , T. Bhaskar , Bioresour. Technol. 2019, 291, 121822. 31352163 10.1016/j.biortech.2019.121822

[128] J. Milovanovic , R. Luque , R. Tschentscher , A. A. Romero , H. Li , K. Shih , N. Rajic , Biomass Bioenergy 2017, 103, 29.

[129] E. Subbotina , A. Velty , J. S. M. Samec , A. Corma , ChemSusChem 2020, 13, 4528. 32281748 10.1002/cssc.202000330

[130] a) Y. Liao , R. Zhong , E. Makshina , M. D'halluin , Y. Van Limbergen , D. Verboekend , B. F. Sels , ACS Catal. 2018, 8, 7861;

[131] H. Tan , S. Rong , R. Zhao , H. Cui , N.‐N. Zhang , Z.‐N. Chen , Z. Li , W. Yi , F. Zhang , Chem. Eng. J. 2022, 438, 135577.

[132] R. Jogi , P. Mäki‐Arvela , P. Virtanen , N. Kumar , J. Hemming , V. Russo , A. Samikannu , T. A. Lestander , J.‐P. Mikkola , J. Energy Inst. 2020, 93, 2055.

[133] B. Zhang , W. Li , T. Zhang , X. Li , A. T. Ogunbiyi , K. Chen , C. Shen , Fuel 2021, 305, 121509.

[134] A. M. Elfadly , I. F. Zeid , F. Z. Yehia , A. M. Rabie , M. M. Aboualala , S.‐E. Park , Int. J. Biol. Macromol. 2016, 91, 278. 27196367 10.1016/j.ijbiomac.2016.05.053

[135] W. Li , X. Dou , C. Zhu , J. Wang , H.‐M. Chang , H. Jameel , X. Li , Bioresour. Technol. 2018, 269, 346. 30195227 10.1016/j.biortech.2018.08.125

[136] a) Y. Zhu , H. Li , J. Xu , H. Yuan , J. Wang , X. Li , CrystEngComm 2011, 13, 402;

[137] D. Gao , A. Duan , X. Zhang , Z. Zhao , E. Hong , J. Li , H. Wang , Appl. Catal. B 2015, 165, 269.

[138] P. Chen , Q. Zhang , R. Shu , Y. Xu , L. Ma , T. Wang , Bioresour. Technol. 2017, 226, 125. 27997866 10.1016/j.biortech.2016.12.030

[139] Y.‐J. Ding , C.‐X. Zhao , Z.‐C. Liu , Bioresour. Technol. 2019, 294, 122097. 31539853 10.1016/j.biortech.2019.122097

[140] Q. Meng , J. Yan , R. Wu , H. Liu , Y. Sun , N. Wu , J. Xiang , L. Zheng , J. Zhang , B. Han , Nat. Commun. 2021, 12, 4534. 34312395 10.1038/s41467-021-24780-8 PMC8313573

[141] X. Dou , X. Jiang , W. Li , C. Zhu , Q. Liu , Q. Lu , X. Zheng , H.‐M. Chang , H. Jameel , Appl. Catal. B 2020, 268, 118429.

[142] S. Kasakov , H. Shi , D. M. Camaioni , C. Zhao , E. Baráth , A. Jentys , J. A. Lercher , Green Chem. 2015, 17, 5079.

[143] D. Son , S. Gu , J.‐W. Choi , D. J. Suh , J. Jae , J. Choi , J.‐M. Ha , J. Ind. Eng. Chem. 2019, 69, 304.

[144] W. Liao , X. Wang , L. Li , D. Fan , Z. Wang , Y. Chen , Y. Li , X. Xie , Energy Fuels 2019, 34, 599.

[145] J. Wu , X. Zhu , Y. Fu , J. Chang , Ind. Eng. Chem. Res. 2022, 61, 3206.

[146] S. Feng , X. Liu , J. Fan , C. Hu , J. H. Clark , Adv. Energy Sustainability Res. 2021, 2, 2100059.

[147] X. Wang , R. Rinaldi , Catal. Today 2016, 269, 48.

[148] J.‐Y. Kim , S. Y. Park , I.‐G. Choi , J. W. Choi , Chem. Eng. J. 2018, 336, 640.

[149] Y. Xu , P. Chen , W. Lv , C. Wang , L. Ma , Q. Zhang , Chin. J. Chem. Eng. 2021, 32, 307.

[150] J. Hu , M. Zhao , B. Jiang , S. Wu , P. Lu , Energy Fuel 2020, 34, 9754.

[151] K. Alper , K. Tekin , S. Karagöz , Biomass Convers. Biorefin. 2019, 9, 669.

[152] S. K. Singh , J. D. Ekhe , Catal. Sci. Technol. 2015, 5, 2117.

[153] S.‐C. Qi , J.‐I. Hayashi , S. Kudo , L. Zhang , Green Chem. 2017, 19, 2636.

[154] S. R. Chia , S. Nomanbhay , M. Y. Ong , K. W. Chew , K. S. Khoo , H. Karimi‐Maleh , P. L. Show , Front. Energy Res. 2021, 9, 769485.

[155] J. Ji , H. Guo , C. Li , Z. Qi , B.o Zhang , T. Dai , M. Jiang , C. Ren , A. Wang , T. Zhang , ChemCatChem 2018, 10, 415.

[156] B. Jiang , J. Hu , Y. Qiao , X. Jiang , P. Lu , Energy Fuels 2019, 33, 8786.

[157] J.‐W. Zhang , Y. Cai , G.‐P. Lu , C. Cai , Green Chem. 2016, 18, 6229.

[158] J. Zhang , H. Asakura , J. Van Rijn , J. Yang , P. Duchesne , B. Zhang , X. Chen , P. Zhang , M. Saeys , N. Yan , Green Chem. 2014, 16, 2432.

[159] J. Zhang , J. Teo , X. Chen , H. Asakura , T. Tanaka , K. Teramura , N. Yan , ACS Catal. 2014, 4, 1574.

[160] Z. Shen , W. Wang , L. Pan , Z. Huang , X. Zhang , C. Shi , J.‐J. Zou , Green Chem. 2023, 25, 7782.

[161] M. Zhao , J. Hu , S. Wu , L. Yang , X. An , P. Yuan , P. Lu , Fuel 2022, 308, 121979.

[162] X. Liu , C. Wang , Y. Zhang , Y. Qiao , Y. Pan , L. Ma , ChemSusChem 2019, 12, 4791. 31453661 10.1002/cssc.201901578

[163] G. Meng , W. Lan , L. Zhang , S. Wang , T. Zhang , S. Zhang , M. Xu , Y. Wang , J. Zhang , F. Yue , Y. Wu , D. Wang , J. Am. Chem. Soc. 2023, 145, 12884. 37249907 10.1021/jacs.3c04028

